# Multiplex viral tropism assay in complex cell populations with single-cell resolution

**DOI:** 10.1038/s41434-022-00360-3

**Published:** 2022-08-23

**Authors:** Choong Tat Keng, Ke Guo, Yu-Chi Liu, Kimberle Yanyin Shen, Daryl Shern Lim, Matthew Lovatt, Heng Pei Ang, Jodhbir S. Mehta, Wei Leong Chew

**Affiliations:** 1 Genome Institute of Singapore, Agency for Science, Technology and Research, 60 Biopolis Street, Singapore, 138672 Singapore; 2 Tissue Engineering and Cell Therapy Group, Singapore Eye Research Institute, Singapore, Singapore; 3 Cornea and Refractive Surgery Group, Singapore Eye Research Institute, Singapore, Singapore; 4 Cornea and External Eye Diseases, Singapore National Eye Centre, Singapore, Singapore; 5 Ophthalmology Academic Clinical Program, Duke-NUS Graduate Medical School, Singapore, Singapore; 6 Synthetic Biology Translational Research Programme, Yong Loo Lin School of Medicine, National University of Singapore, Singapore, 117596 Singapore

**Keywords:** Genetic vectors, Gene therapy

## Abstract

Gene therapy constitutes one of the most promising mode of disease treatments. Two key properties for therapeutic delivery vectors are its transduction efficiency (how well the vector delivers therapeutic cargo to desired target cells) and specificity (how well it avoids off-target delivery into unintended cells within the body). Here we developed an integrated bioinformatics and experimental pipeline that enables multiplex measurement of transduction efficiency and specificity, particularly by measuring how libraries of delivery vectors transduce libraries of diverse cell types. We demonstrated that pairing high-throughput measurement of AAV identity with high-resolution single-cell RNA transcriptomic sequencing maps how natural and engineered AAV variants transduce individual cells within human cerebral and ocular organoids. We further demonstrate that efficient AAV transduction observed in organoids is recapitulated in vivo in non-human primates. This library-on-library technology will be important for determining the safety and efficacy of therapeutic delivery vectors.

## Introduction

Adeno-associated viruses (AAVs) are medically and commercially attractive gene delivery vectors due to the recent successes in FDA and EMA approvals for AAV-based gene therapies, as exemplified by Glybera for the treatment of lipoprotein lipase deficiency, Luxturna for the treatment of inherited retinal disease, and Zolgensma for the treatment of paediatric spinal muscular dystrophy [1–5]. The therapeutic application of AAVs span from targeting small tissues in the eye to systemic distribution in the muscles as well as difficult-to-access systems such as the nervous system and vasculature [1]. This versatility is enabled by the ability to manipulate the AAV protein capsid sequence, which in turns changes the serotype and confers preferential tropism towards desired tissues. While considerable efforts have been devoted to identifying optimal capsid proteins for successful therapy, early studies comparing the performance of different AAV serotypes are often of low-throughput and costly. A first limitation is that each cell line or animal is usually only transduced by a single AAV serotype. Hence, evaluating multiple different serotypes would require an increase in independent replicates. This is in part because readouts employed for transduction efficiency assays tend to be non-multiplexable, such as quantification by immunohistology or fluorescence reporter proxy [6–10], which means that each sample could only be tested with a single candidate vector. A second limitation is that the sensitivity of transduction assays tends to require aggregation across many cells and vector copies, and hence the resolution is limited to the tissue level instead of the often-required cellular level. Such single-plex approaches limit comparison to only a small handful of AAV serotypes in a similarly small number of target cells or tissues. In recent years, transduction assays of higher throughput have been devised by harnessing sequencing as a readout for transduction efficacy, where multiplex libraries of AAVs bearing nucleotide barcodes are administered to the target cells or tissues and the best-performing AAV serotype are identified by sequencing the nucleotide barcodes [11–15]. However, the techniques employed so far have been limited to bulk tissues, which do not offer the resolution needed to profile how efficiently or specifically each AAV serotype transduces specific subset(s) of cells within a complex tissue population [16–19]. More recently, some studies have explored the use of single-cell technology and AAV barcoding techniques to match AAV tropism to different cell populations. But these required separate preparation steps for the single cell transcriptomes and the viral barcodes libraries, and a complex bioinformatics pipeline for processing the cell identity, the viral vector barcode information, and then mapping the viral barcodes to the cell identity for further tropism analysis [20, 21].

In this study, we developed an integrated technology pipeline that enables multiplex measurement of AAV transduction efficiency and specificity for each cell type within a heterogeneous population by using a single library preparation and bioinformatics workflow. We barcoded AAV serotypes and applied this AAV library on complex mixtures of cell types (ie. organoids), conducted single-cell sequencing [22–26] to identify cell types and the AAV barcodes in each cell, and deconvoluted the data into matrices of AAV serotype versus human cell types directly in the Cell Ranger software and results can be visualized in Seurat. We applied this technology in human organoids, which can recapitulate certain structural and cellular complexity of the human brain and eye, and identified how efficiently and specifically each AAV serotype transduces individual cell types found within the organoids [27–31]. To show the robustness of the multiplex AAV screen, we select to validate the tropism of AAV6, which is identified as the best performing serotype for corneal epithelial cells, in a marmoset model and demonstrated the effectiveness of transduction of AAV6-SpCas9 in the non-human primate corneal epithelial cells. This technology enables a more comprehensive interrogation of delivery vector biodistribution that will impact evaluating the safety and efficacy profiles of the therapeutic product.

## Materials and methods

### Organoids culture and condition

Briefly, the cerebral and ocular organoids were cultured in mTeSR1 medium (Stem Cell Technologies, cat. no. 85850). Human ES cell (Wicell H1 WA01 and H9 WA09) were treated by accutase to generate single cells. Then, 4000 cells were plated in each well of a V-bottom 96-well plate (Sematec Pte Ltd Code: 1009985) with low concentrations of basic fibroblast growth factor (bFGF 4 ng/ml) and 20 µM/ml Rho-associated protein kinase (ROCK) inhibitor (Y27632 Stem Cell). The next day, Embryonic Bodies (EB) were transferred into in low-attachment 96 well U-bottom plate with hESC medium (For 500 ml of medium, combine 400 ml of DMEM-F12, 100 ml of KOSR, 15 ml of ESC-quality FBS, 5 ml of GlutaMAX, 5 ml of MEM-NEAA and 3.5 μl of 2-mercaptoethanol) for cerebral organoid, Differentiation Medium DM (DMEM/F12, 4% knockout serum replacement (KOSR), 4% fetal bovine serum (ESC-quality FBS), 1× non-essential amino acids (NEAA), 1× Glutamax, 1× Pen-Strep. Filter it using a vacuum-driven 0.2-μm filter unit) for ocular organoid. EB were fed every other day for 6 days and then changed into neural induction media for cerebral organoid and into retinal differentiation medium (RDM: DM + 2% B27) for ocular organoid for the next 4 days. After the EB undergone neuro-ectodermal differentiation, they are transferred to Matrigel (Growth factor–reduced Matrigel, Bio-Lab 354230). Making matrigel in 1:1 dilution with cerebral organoid differentiation medium or corneal differentiation medium (CDM). 50 µl of matrigel is added to each well and incubated for 30 min in a 37 °C incubator, followed by adding 100ul cerebral organoid differentiation medium with B27 (−) Vitamin A to each well and cultured for 48 h. After 2–3 days, the aggregates (organoids) were transferred to 6-well clear flat-bottom ultra-low attachment plates. After 4 days of static culture with cerebral organoid differentiation medium with B27 (−) Vitamin A, the embedded organoids were transferred to an orbital shaker at 80 rpm within 37 °C, 5% CO_2_ incubator for long-term culture with cerebral organoid differentiation medium with B27 (+) Vitamin A.

### AAV plasmid cloning and virus production

The barcoded eGFP plasmids were constructed by introducing a short sequence TAATAAATCGATCGNNNNNNNN after the eGFP transgene stop codon in the plasmid backbone pZac2.1-CMV-eGFP.rgb, a gift from Luk Vandenberghe. Primers with overhanging barcode were designed for first round PCR to generate barcoded eGFP fragments that terminates at ITR sequences. A second round of nested PCR amplify shorter fragments of barcoded eGFP which are digested with restriction enzyme NheI and BamHI. Digested fragments are ligated with the vector backbone which is digested using the same restriction enzymes. The sequences of the clones were checked by Sanger sequencing. The representing barcodes for each AAV serotype are shown in Table S1. The serotype-specific pAAV-RepCap plasmids were constructed by cloning in the Cap genes from the different serotypes into the pAAV-RepCap backbone using Gibson assembly. The different serotypes Cap genes were ordered as gene blocks (IDT) and cloned into HindIII/PmeI-digested pAAV-RepCap backbone via Gibson assembly to construct the pAAV-RepCap with the different serotypes Cap genes. AAV viruses from different serotypes each bearing its own barcode were produced as per standard protocol [32]. Briefly, AAV were packaged via a triple transfection of 293AAV cell line (Cell Biolabs AAV-100) that were plated in a HYPERFlask ‘M’ (Corning) in growth media consisting of DMEM + glutaMax+pyruvate+10%FBS (Thermo Fisher), supplemented with 1X MEM non-essential amino acids (Gibco). Confluency at transfection was between 70–90%. Media was replaced with fresh pre-warmed growth media before transfection. For each HYPERFlask ‘M’, 200 μg of pHelper (Cell Biolabs), 100 μg of pRepCap [encoding capsid proteins for different serotypes], and 100 μg of pZac-CASI-GFP (barcoded) were mixed in 5 ml of DMEM, and 2 mg of PEI “MAX” (Polysciences) (40 kDa, 1 mg/ml in H_2_O, pH 7.1) added for PEI: DNA mass ratio of 5:1. The mixture was incubated for 15 min, and transferred drop-wise to the cell media. The day after transfection, media was changed to DMEM + glutamax+pyruvate+2%FBS. Cells were harvested 48–72 h after transfection by scrapping or dissociation with 1×PBS (pH7.2) + 5 mM EDTA, and pelleted at 1500 g for 12 min. Cell pellets were resuspended in 1–5 ml of lysis buffer (Tris HCl pH 7.5 + 2 mM MgCl + 150 mM NaCl), and freeze-thawed 3× between dry-ice-ethanol bath and 37  °C water bath. Cell debris was clarified via 4000 g for 5 min, and the supernatant collected. The collected supernatant was treated with 50 U/ml of Benzonase (Sigma-Aldrich) and 1 U/ml of RNase cocktail (Invitrogen) for 30 min at 37  °C to remove unpackaged nucleic acids. After incubation, the lysate was loaded on top of a discontinuous density gradient consisting of 6 ml each of 15%, 25%, 40%, 60% Optiprep (Sigma-Aldrich) in an 29.9 ml Optiseal polypropylene tube (Beckman-Coulter). The tubes were ultra-centrifuged at 54000 rpm, at 18  °C, for 1.5 hr, on a Type 70 Ti rotor. The 40% fraction was extracted, and dialyzed with 1×PBS (pH 7.2) supplemented with 35 mM NaCl, using Amicon Ultra-15 (100 kDa MWCO) (Millipore). The titer of the purified AAV vector stocks were determined using real-time qPCR with ITR-sequence-specific primers and probe [33], referenced against the ATCC reference standard material 8 (ATCC).

### In vitro transduction of organoids

AAV serotypes pool was created by pooling each AAV serotype at 1 × 10^10^ vg, giving a final viral copy of 9 × 10^10^ that is used for the transduction of organoids in each well of a 24-well plate. AAV1, 2, 6, 7, 8, 9, rh10, DJ and Anc80 serotypes were used for the pooling. Organoids were transduced for 7–10 days before harvesting for sequencing, fluorescence imaging, and histochemistry.

### Immunofluorescence histochemistry

Organoids and marmoset’s corneas were fixed in 4% paraformaldehyde for 4 h at 4 °C followed by washing in PBS three times for 15 min. Organoids and marmoset’s corneas were allowed to sink in 30% sucrose overnight and then embedded in OCT and cryosectioned at 12 µm. Marmoset’s corneas were vertical embedded and sectioned. Sections were permeabilized in 0.2% Triton X-100 in PBS and blocked in block buffer (2% BSA 5% fetal bovine serum) for 1 h at room temperature. Sections were subsequently incubated with the indicated primary antibodies at a 1:100 dilution in block buffer at 4 °C overnight. Secondary antibodies used were donkey Alexa Fluor 488, 568 and 647 conjugates (Invitrogen, 1:1000). After staining with 4′,6-diamidino-2-phenylindole (DAPI) (Sigma-Aldrich) in PBS for 5 min, slides were mounted in Vectashield anti-fade reagent (Vector Laboratories). Confocal imaging was performed with Leica TCS SP8 DLS LightSheet microscope. Primary antibodies: PAX6 (rabbit, ab5790), CHX10 (rabbit, ab133636), ZO-1 (mouse, ThermoFisher ZO1-1A12), MAP2 (chicken, ab5392), S100β (rabbit, ab52642), RAX (rabbit, ab23340), CD31 (mouse, ab23340), aSMA (rabbit, ab5694), DAPI (49,6-diamidino-2-phenylindole), ThermoFisher D1306, NeuN (mouse, Sigma-Aldrich MAB377), AAV (rabbit ab45482), Cas9 (Sp)(E7M1H) XP^®^ (rabbit, CST #19526, CK3 (mouse, ab68260).

### Amplicon barcode sequencing and analysis

AAV serotypes pool were checked for percentage distribution by NGS library preparation and the correction factor was used for re-adjusting the pooling of the AAV serotypes for subsequent experiment. Custom primers were designed for a first round of 20-cycle PCR of the target site containing the AAV barcodes as shown in Table S1. Target bands are extracted using gel extraction and a second round of 15-cycle PCR were used for adding P5 and P7 adapter sequences to the enriched fragments and the final libraries were cleaned up by gel extraction. Primers used for library construction are shown in Table S2. Library concentrations were determined using a Qubit dsDNA HS kit (Agilent). NGS sequencing were carried out on the MiSeq using 2 × 75 bp PE run with 20% PhiX spike-in. An in-house python script was utilized to search for the 8 unique nucleotide barcode sequences representing each serotype within the MiSeq FASTQs generated from the MiSeq run of the amplicon libraries and the total count was tabulated for each barcode sequence for each sample (Refer to PythonScriptforBulkAnalysis package). Transduced organoid samples were harvested as single cells and processed through the 10X chromium machine for cell barcoding of the transcripts. The total cDNA was purified via the 10X workflow and 5ul was aliquoted for custom bulk-sequencing as described above. The rest of the cDNA were used to proceed with the remaining 10X workflow for single-cell sequencing.

### Single cell sequencing and RNA transcriptomic analysis

Samples were prepared as indicated in the 10X Genomics Single Cell 3′ v2 Reagent Kit user guide. The single-cell libraries were prepared by following the manufacturers’ protocol followed by sequencing on an Illumina HiSeq4000 flow cell. The sequencing data were processed by the standard Cell Ranger pipeline using the modified gtf and genome manifest files. Briefly, the samples were washed twice in PBS (Life Technologies) + 0.04% BSA (Sigma) and re-suspended in the same solution. Sample viability was assessed using Trypan Blue (Thermo Fisher) under a light microscope. Following viability counting, the appropriate volume for each sample was calculated for a target capture of 10,000 cells and loaded onto the 10x Genomics single-cell-A chip along with other reagents and barcoded beads by following the protocol guide. The chip is then loaded onto a 10X Chromium machine for droplet generation and samples were transferred onto a pre-chilled strip tube (Eppendorf), and reverse transcription was performed using a 96-well thermal cycler (Thermo Fisher). After the reverse transcription, cDNA was recovered using Recovery Agent provided by 10X Genomics, followed by Silane DynaBead clean-up (10X Genomics). Purified cDNA was amplified for 12 cycles before being cleaned up using SPRI-select beads (Beckman). Samples were diluted 4 times in water and ran on a Bioanalyzer (Agilent Technologies) to determine cDNA concentration. cDNA libraries were then prepared following the Single Cell 3′ Reagent Kits v2 user guide with appropriate PCR cycles based on the cDNA concentration as determined by the bioanalyzer. The molarity of the single cell libraries was calculated based on their library sizes as measured using a bioanalyzer (Agilent Technologies) and using the KAPA qPCR quantification (KAPA) method on a qPCR cycler (Roche). Samples were normalized to 10 nM before sequencing. Each organoid sample was sequenced on a full lane on a HiSeq 4000 with the following run parameters: Read 1–26 cycles, read 2–98 cycles, index 1–8 cycles. Using the FASTQ files from each sample, the standard Cell Ranger Count command pipeline was performed for transcripts read alignment, UMI counting, and clustering (Amazon Web Services via the Ronin cloud platform). Raw data were processed using standard Cell Ranger transcriptomics command, while using modified genome reference file and the modified gtf file. For command lines for the modification of gtf file and genome reference file to include the barcoded GFP sequences, refer to Supplementary Data S1. Command lines for cell count using the modified files are also shown in Supplementary Data S1. Finally, upon successful cellranger count run, the output files can be used for single cells visualization and analysis on the Seurat software (v4.0.1).

### Single cell AAV tropism analysis

For the purpose of parallel sequencing of the AAV barcodes in single cells along with the RNA transcripts, the human genome reference file and the genome transcript file (gtf) were modified (Supplementary Data S1). Briefly, the names and barcodes of each AAV serotypes are manually included into both files that will be used for the execution of the Cell Ranger Count command pipeline in order to include the AAV barcode transcripts into the read alignment, UMI counting, and clustering. To include the AAV barcode representation in the genome reference file, the command line “>GFP1 TAAATCGATCGNNNNNNNN” is included for each barcode, where the 8Ns represent a unique 8 nucleotide barcode sequence. The command line “GFP me exon 1 19 - + - gene_id “GFP1”; transcript_id “GFP1”” was included in the genome transcript file for each AAV barcode representation added to the genome reference file (Supplementary Data S1). After processing by Cell Ranger to obtain the gene count matrices, further computations were carried out in R (version 4.0.4). Quality control, normalization, PCA, clustering and downstream analysis were performed with Seurat (version 4.0.1). Briefly, cells with less than 200 RNA features or more than 10% mitochondrial reads were removed from analysis. The dimensions of the final data matrices are 17,022 features across 5741 cells (Ocular dataset) and 20281 features across 15,335 cells (Cerebral dataset). After normalization, PCA was performed with the top 2000 most variable features, followed by Louvain clustering with a resolution of 0.3–0.4 to achieve reasonable distinction of cell type clusters, and dimensional reduction using tSNE. Plots and figures were assembled using Seurat and the ggpubr (version 0.4.0) package. To determine the transduction specificity of a specific viral vector against a specific cell niche relative to other cell niches, we calculated the frequencies with which the presence of that specific viral vector is detected in the cells of the specific cell niche, against the frequencies with which the presence of the same specific viral vector is detected in the cells of other specific cell niches.

### Marmoset animal work

A total of 2 marmosets were used in this study. The animals were sedated with 10–15 mg/kg Ketamine and 4–5% isoflurane, and then intubated with an appropriate endotracheal tube by an experienced veterinarian. The eyes were cleaned with 2.5% povidone-iodine and draped, before a small-sized eyelid speculum was inserted. A single injection of 30 μL of AAV6-SpCas9 (Total 1.9 × 10^11^ vgs) suspension was injected into the anterior chamber with a 30-gauge needle without touching the lens, iris, or other ocular tissue. After the injection, a Weck-Cel eye sponge was applied to the incision site for 1 minute to prevent bleeding and AAV suspension efflux. The anterior chamber was left formed with no aqueous leak. All the eyes received topical tobramycin ointment (Alcon, Fort Worth, TX, USA) once at the end of the procedure. Three months after injection, the animals were euthanized under general anesthesia with intravenous injection of pentobarbital (60–100 mg/kg). After enucleation of the eyeballs, the corneas were harvested. A small limbal incision was made with a diamond knife, and the cornea was excised 360° alongside the limbus with corneal scissors. There was no lens-cornea touch, damage of iris and lens tissue, or excessive corneal curvature deformation during the harvesting. The excised cornea was kept moist with normal saline. Subsequently, DM peeling was performed manually. The corneal tissue was placed onto an anatomically shaped bowel, of a Coronet corneal trephine vacuum punch (Network Medical Products, North Yorkshire, UK). During the DM peeling, the corneal tissue was submerged in normal saline solution to minimize the surface tension on the tissue and allow the DM to settle back onto the corneal stroma. The DM was then grasped with two fine non-toothed Kelman forceps and was slowly and gently stripped completely away from the stroma from the edge towards the center. The remaining tissue (with epithelial & stroma) is then cut into 4 quarters for different analytical procedures. One quarter was used for DNA extraction, one quarter for RNA extraction and the remaining two quarters for immunohistochemistry. No adverse event was noted.

### qPCR quantification for marmoset tissues

Total DNA were extracted from a quarter of the harvested marmoset cornea using QuickExtract (Lucigen) by following manufacturer’s protocol. The titer of the purified AAV vector stocks were determined using real-time qPCR with ITR-sequence-specific primers and probe (Table S5), referenced against the ATCC reference standard material 8 (ATCC). Total RNA were extracted from a quarter of harvested marmoset cornea using the RNeasy universal plus mini kit (Qiagen) by following manufacturer’s protocol. cDNA was synthesized using Superscript III (Thermo Fisher) and polyT primer, and subsequently quantified using real-time qPCR with Cas9-sequence-specific primers and probe (Table S5). For normalization, GAPDH was quantified using real-time qPCR with marmoset GAPDH-sequence-specific primers and probe (Table S5).

## Results

### Study design

**Fig. 1:**
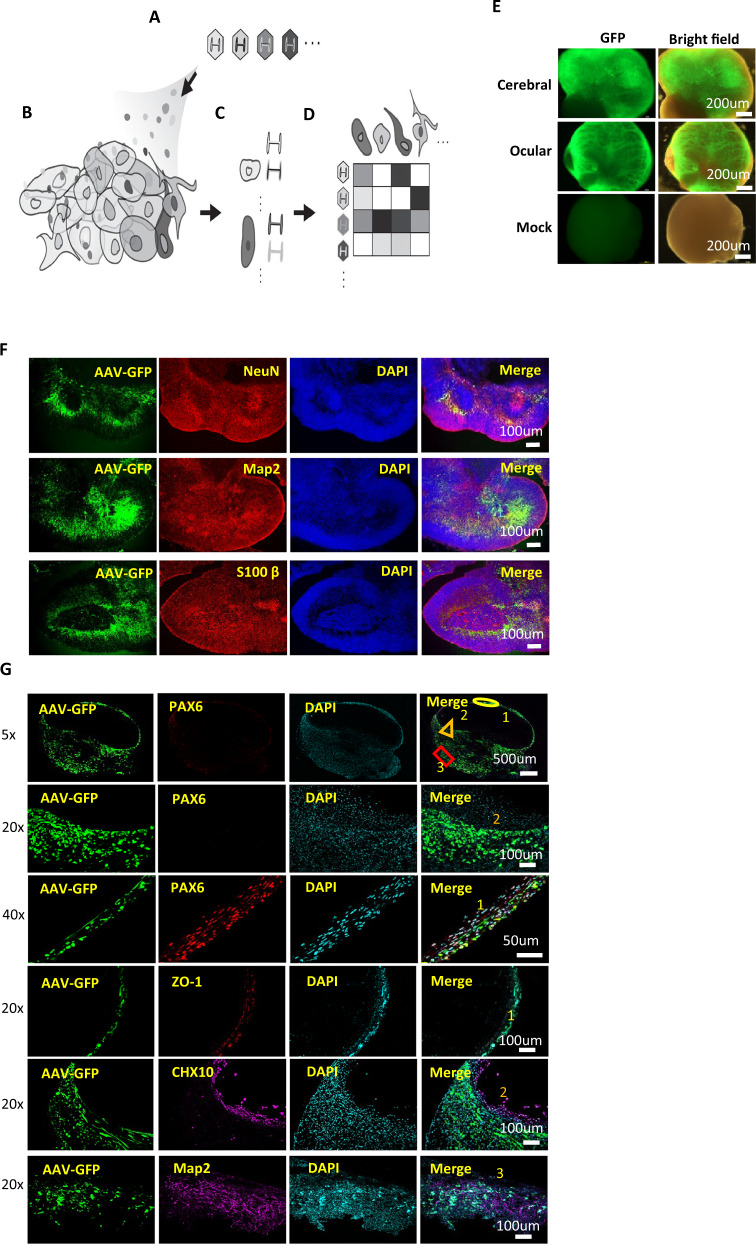
Framework for multiplex AAV tropism analysis at single cell resolution for pooled AAV transduced human ocular and cerebral organoids. **A** AAV capsid serotypes are individually packaged with uniquely identifiable genomes containing AAV barcode sequences. **B** Library of AAV variants transduce a heterogenous population of cells (e.g. organs, tissues, organoids, admixtures). **C** In single-cell sequencing, nucleic acids (which can include RNA, DNA) within each cell are tagged by a unique cell-specific single-cell-sequencing nucleotide tag, then sequenced, and each cell is identified by its RNA transcriptome and/or DNA genome. **D** The matrix of which cell identity is being transduced by which AAV serotype can be created by matching the AAV variants with their respective transduced cells through the matching of their cell-specific single-cell-sequencing nucleotide tags. **E** Gross Morphology of cerebral and ocular organoids transduced with AAV serotypes pool. Barcoded GFP-AAV-Pool (1 × 10^10^ vg/per serotype) expressing eGFP were used for transduction of cerebral and ocular organoids for 7 days. Low magnification microscopy showed GFP-positive signals in cells within most regions. *Mock* indicates negative control of un-transduced organoids. **F** Immunofluorescence staining of cellular markers and GFP protein for identification of cell types transduced by the AAV serotypes pool in cerebral organoid. **G** Immunofluorescence staining of cellular markers and GFP protein for identification of cell types transduced by the AAV serotypes pool in ocular organoid. PAX6 - ocular epithelial or endothelial cells. CHX10 -specification and morphogenesis of the sensory retina. ZO-1- corneal endothelia marker. MAP2 –neuronal marker. DAPI marks nuclei.

In this study, we aim to provide a new framework for assessing multiplex viral tropism in complex tissues in a high-throughput manner and at single-cell resolution. To accomplish this aim, we first generated panels of AAV serotypes where the AAV cargo is uniquely differentiable from each other. Specifically, each AAV serotype contains an eGFP transgene that is barcoded by a unique 8 bp sequence at its 3’ end prior to the polyadenylation tail sequence (Supplementary Fig. S1A). The unique barcode length can be arbitrarily extended to enable an exponential library size. AAVs were produced from these barcoded packaging plasmids and the pooled AAVs were then used to transduce heterogeneous populations of cells within human ocular and cerebral organoids. Following transduction and cargo expression within transduced cells, the organoids were then dissociated for single-cell sequencing, so as to identify the cell type and the AAV barcodes that transduced the particular cell (Fig. 1A–D). Modifications made to the genome reference file and genome transcript file allowed for the AAV barcoded transcripts to be aligned and analyzed together with the RNA transcriptomics data for the assignment and visualization of each AAV serotype transcript to individual cells using Seurat (v4.0.1) (refer to Materials and Methods).

### Barcoded AAVs transduce diverse tissue subtypes in human ocular and cerebral organoids

We reasoned that human ocular and cerebral organoids serve as models that represent the complexity of human tissues comprising multiple cellular subtypes. The organoids were cultured by differentiating H1 and H9 lineage of human ES cells on petri dishes for 6 weeks (Supplementary Fig. S2A), following which the ocular organoids were characterized by immuno-staining for common ocular tissue cellular markers [34–37] S100β, PAX6, CHX10, RAX, CD31 and αSMA (Supplementary Fig. S2B) and the cerebral organoids were characterized by immuno-staining with common neural tissue cellular markers [27, 28, 30] S100β, NeuN and Map2 (Supplementary Fig. S2C). Both the ocular and cerebral organoids express different cellular markers in distinct cellular layers, indicating heterogenous tissue subtypes within the organoids. The barcoded AAV pools (1 × 10^10^ vg/per serotype) were then administered to the cerebral and ocular organoids. Culturing these organoids for a further 7 days resulted in strong GFP-positive signals in cells within most regions of the organoids indicating transduction and expression of the GFP cargo common among the pooled AAVs (Fig. 1E). Co-localization of eGFP with several different cellular markers also confirmed that the pooled AAVs transduced diverse tissue subtypes within the human ocular and cerebral organoids (Fig. 1F, G).

### Single cell RNA transcriptomics clustering and assignment of AAV barcoded mRNA transcripts in transduced ocular and cerebral organoids at single cell resolution

**Fig. 2:**
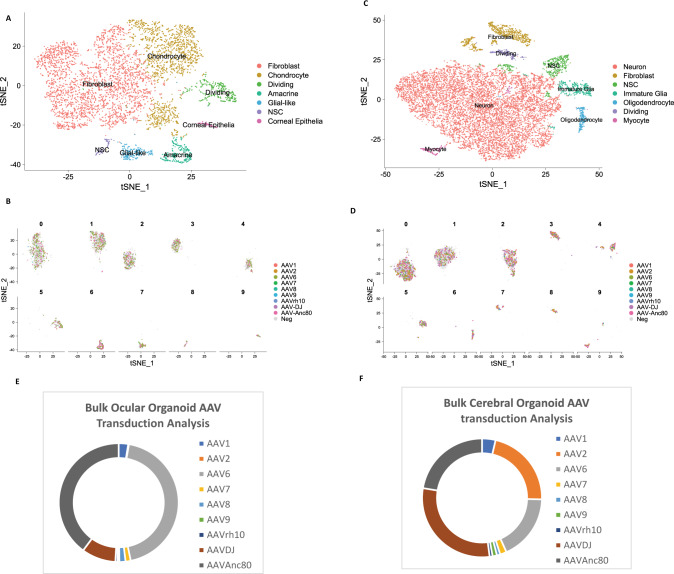
Single cell assignment of barcoded AAV transcripts for human ocular and cerebral organoids versus bulk analysis of AAV tropism. **A** The t-Stochastic Neighbor Embedding (t-SNE) plot of 5849 cells from the human ocular organoid derived from H1 human ES cells separated into 7 distinct cell types based on major gene expression markers. **B** t-SNE plots showing individual cells in the ocular organoid transduced with different AAV serotypes (each serotype represented by one color), in each of the 8 clusters. **C** The t-Stochastic Neighbor Embedding (t-SNE) plot of 15466 cells from the human cerebral organoid derived from H1 human embryonic stem cells, separated into 7 distinct cell types based on major gene expression markers. **D** t-SNE plots showing individual cells in the cerebral organoid transduced with different AAV serotypes (each serotype represented by one colour), in each of the 8 clusters. **E** Bulk analysis of the transduced ocular organoid by amplicon-sequencing on MiSeq sequencer. Results from bulk sequencing analysis is in agreement with the single-cell analysis plots processed with Cell Ranger pipeline (refer to Table S4), indicating that this assay enables accurate measurement of AAV tropism with single-cell resolution, beyond traditional bulk sequencing approach. **F** Bulk analysis of the transduced cerebral organoid by amplicon-sequencing on MiSeq sequencer. Results from bulk sequencing analysis using custom Python script is in agreement with the single-cell analysis plots processed with Cell Ranger pipeline (refer to Table S4), indicating that this multiplex screening platform enables accurate measurement of AAV tropism with single-cell resolution, beyond traditional bulk sequencing approach.

After the human ocular organoids were transduced with the AAV libraries as described above, they were trypsinized into single cells as input for single-cell library preparation and sequencing (Materials and Methods). For the transduced ocular organoid, the transcriptomes of 5849 cells within the organoid were profiled with mean reads per cell at 122,688 and median genes per cell at 1022. Using Seurat t-SNE clustering, we were able to define 7 cell types for 10 cell clusters within the ocular organoid based on their transcriptomic profile (Figs. 2A, S3A and Supplementary Data S2). Each cell type was annotated based on major gene expression markers of known cell types in ocular tissues (Supplementary Fig. S3C). Minor known gene markers in gene expression profiles for each cluster were also checked to confirm that the correct annotation was assigned for the cell cluster (Supplementary Data S2). Figure 2B shows a summary of all assignment of the AAV transcripts with each colour representing one AAV serotype, at single cell resolution. For more detailed analysis, the plot can be broken down to visualize individual serotype for each cluster (Supplementary Fig. S4). Cell cluster 0 to 9 were assigned cell identities of “Fibroblast”, “Chondrocyte”, “Fibroblast”, “Fibroblast”, “Chondrocyte”, “Dividing”, “Amacrine”, “Glial-like”, “Neural stem cells” and “Corneal Epithelia” respectively (Fig. 2B).

To demonstrate that the methodology is easily applied on different complex tissues, we also conducted the same single-cell tropism assay on human cerebral organoids, which contain different populations of cell types compared to the ocular organoids. For the transduced cerebral organoid, single-cell sequencing profiled the transcriptomes of 15,466 cells within the organoid with mean reads per cell at 23,315 and median genes per cell at 902. Similarly, using Seurat t-SNE clustering, we were able to define 7 cell types for 10 clusters of cells within the cerebral organoid based on their transcriptomic profile (Figs. 2C, S3B and Supplementary Data S3). Similarly, cell type annotation was performed for the cerebral organoid using gene markers and expression profile (Supplementary Fig. S3D, Supplementary Data S3). Figure 2D shows a summary of the assignment of all the AAV transcripts with each colour representing one AAV serotype, at single cell resolution. For more detailed analysis, the plot can be broken down to visualize individual serotype for each cluster (Supplementary Fig. S5). Cell cluster 0 to 9 were assigned cell identities of “Neuron”, “Neuron”, “Neuron”, “Fibroblast”, “Neural stem cells”, “Immature glial”, “Oligodendrocytes”, “Fibroblast”, “Dividing cell” and “Myocytes” respectively (Fig. 2D).

Next, we compared the multiplex tropism assessment technology to bulk sequencing of the GFP barcodes, which is a method commonly used by recent studies to examine AAV transduction in bulk tissues. Data from bulk sequencing of ocular organoid is in concordance with the single-cell sequencing data in silico*-*aggregated across cells, with AAV-Anc80, AAV6 and AAV-DJ being the top 3 AAV serotypes that most efficiently transduce the ocular organoid in bulk or in aggregate among single-cells (Table S3 and Fig. 2E). Similarly, the data from bulk sequencing of the cerebral organoid also aligns with the single cell sequencing data with AAV2, AAV6, AAV-DJ and AAV-Anc80 as the top 4 AAV serotypes that can most efficiently transduce the cerebral organoid (Table S4 and Fig. 2F). Hence, the single-cell technology is concordant with the recent tropism assays utilising bulk sequencing.

**Fig. 3:**
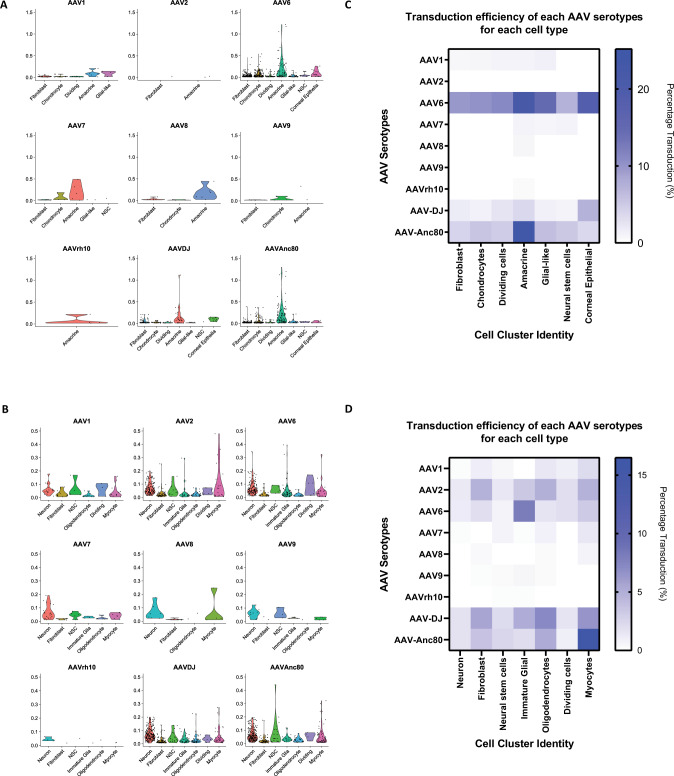
High-throughput AAV tropism measurement and analysis for human ocular and cerebral organoids. **A** AAV cell cluster tropism in transduced human ocular organoid. Data represent the cell counts that are transduced with each AAV serotype across the different cell types within the human ocular organoid. **B** AAV cell cluster tropism in transduced human cerebral organoid. Data represent the cell counts that are transduced with each AAV serotype across the different cell types within the human cerebral organoid. **C** The transduction efficiency of each AAV serotype for each cell type within the human ocular organoid is visualized as the percentage of cells transduced in a heat map. Using this method, this assay enables identification of (i) the most efficient AAV serotype for each cell cluster and (ii) the most specific AAV serotype for the target cell type of choice (i.e. lowest transduction of other non-desired cell types). **D** The transduction efficiency of each AAV serotype for each cell type within the human cerebral organoid is visualized as the percentage of cells transduced in a heat map plot. Using this method, this assay enables identification of (i) the most efficient AAV serotype for each cell cluster and (ii) the most specific AAV serotype for the target cell type of choice (i.e. lowest transduction of other non-desired cell types).

Importantly, the single-cell technology offers a much higher resolution than that afforded by bulk sequencing. By extracting the read counts of the different AAV serotypes transcripts in each cell cluster, we were able to visualize the relative transduction efficiency of each AAV serotype across heterogeneous cell types within the organoids (Fig. 3A, B). The single cell analysis software, Seurat, implements a global-scaling normalization method “LogNormalize” that normalizes the gene expression measurements for each cell by the total expression, multiplies this by a scale factor (10,000 by default), and log-transforms the result. For ocular organoid, when normalized against total expression, AAV-6 and AAV-DJ are identified as the most efficient serotype for targeting corneal epithelial cells (KRT14^hi^), while AAV6 is the most efficient serotype transducing glial-like cells (APOE^hi^) (Fig. 3A, C). In addition, AAV6 and Anc80 were identified as the most efficient transducer for amacrine cells (GJD2^hi^) while AAV6 is the most efficient vector for fibroblast (COL1A1^hi^), chondrocytes (COL2A1^hi^), neural stem cells (SOX2^hi^) and dividing cells (CDK1^hi^) (Fig. 3A, C). Similarly, for the cerebral organoid, when normalized against total expression, AAV2 and AAV-DJ were identified as serotypes that can efficiently transduce fibroblast (COL1A1^hi^) and oligodendrocytes (PLP^hi^) while AAV6 is the most efficient in transducing immature glial cells (CST3^hi^). In addition, the result suggests AAV-Anc-80 is the most efficient serotype for transducing myocytes (MYOG^hi^) (Fig. 3B, D). Neuron cells (GAP43^hi^), neural stem cells (SOX2^hi^) and dividing cells (CDK1^hi^) are permissible to serotypes AAV2, 6, DJ or Anc80 (Fig. 3B, D).

To validate the robustness of the technology, we confirmed that modification of the Cell Ranger pipeline does not affect the clustering of the cell populations via comparing the same data with or without the AAV and eGFP barcodes included in the reference file (Fig. S6A, C). In contrast, the transduced organoid and the non-transduced organoid displayed segregated cell clusters (Fig. S6B, D). This could either be due to AAV transduction affecting the cell compositions within organoids or, more likely, the asynchrony of organoids as expected from variability in cell type and development status inherent in separate bulk populations. Nevertheless, relative transduction efficacy of AAV vectors for each cell type in the organoid is directly associated with these individual cell types as determined via single-cell transcriptomes, highlighting how single-cell sequencing here can reduce inaccuracies associated with assays on bulk populations and across multiple samples. To test the reproducibility of the technology, we repeated the pipeline using additional organoids harvested at different time-point (8 weeks instead of 6 weeks) or cultured from different ES cell line. Importantly, while the distribution of cell types can change according to the organoids being cultured from different ES cells or harvested at different time-points (Fig. S7), the tropism and performance of AAV serotypes are consistent in the individual cell types across samples, for example, the corneal epithelial subpopulation of cells are always transduced efficiently by AAV6 and AAV-DJ (Figs. 2A and S7A).

These results show that the single-cell AAV tropism assay identifies different AAV serotypes with preferential tropism towards each subset of human cell types within the ocular or cerebral organoids.

### In vivo validation of AAV6 tropism for ocular epithelial cells in marmoset model

**Fig. 4:**
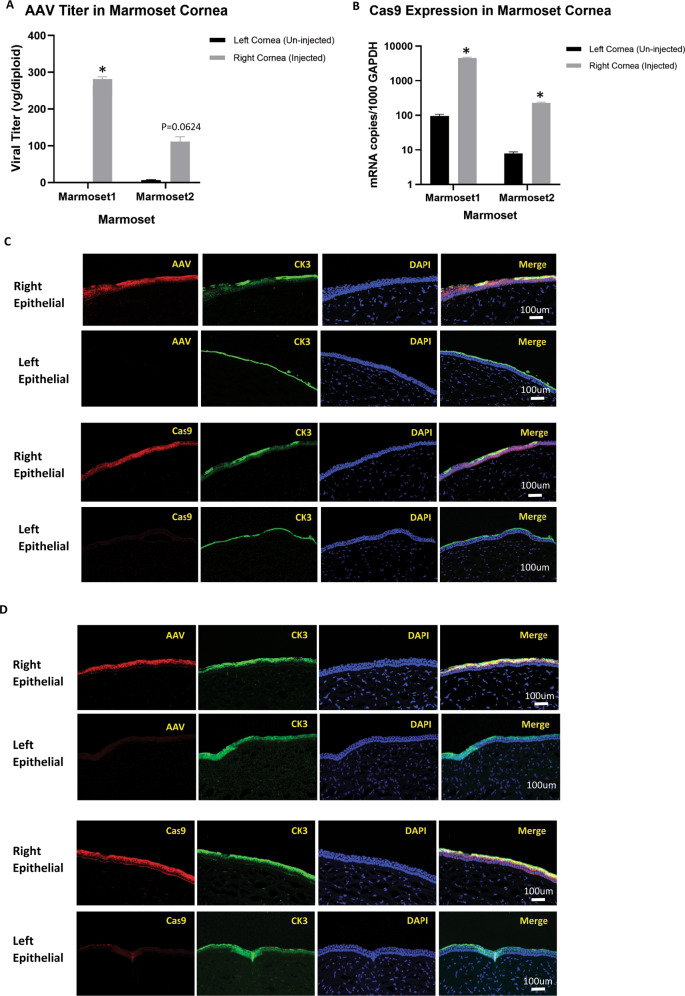
In vivo evaluation of AAV6-SpCas9 transduction of ocular epithelial tropism in marmoset model. **A** AAV virus titer in the marmoset cornea epithelial tissue was quantified by qPCR using specific probe and primers and normalized to diploid copies. Comparison between control left cornea and treated right cornea was analyzed by Student *t* test (two-tailed) using the software GraphPad Prism 9. *p < 0.05. **B** Cas9 mRNA expression level was quantified in extracted RNA from the marmoset cornea epithelial tissue and normalized using GAPDH. Comparison between control left cornea and treated right cornea was analyzed by Student *t* test (two-tailed) using the software GraphPad Prism 9. *p < 0.05. **C** Immunohistochemistry staining of marmoset1 cornea epithelial tissue. Presence of AAV viral antigen (first row) and SpCas9 cargo protein (third row) was detected in the right cornea epithelial layer (co-stained by CK3 antibody) but absent in the untreated left cornea epithelial layer (second and fourth row). **D** Immunohistochemistry staining of marmoset2 cornea epithelial tissue. Presence of AAV viral antigen (first row) and SpCas9 cargo protein (third row) was detected in the right cornea epithelial layer (co-stained by CK3 antibody) but absent in the untreated left cornea epithelial layer (second and fourth row).

To test the robustness of the assay, we select to validate the result of AAV tropism for ocular epithelial cells using a marmoset model. In the multiplex AAV tropism single-cell assay with ocular organoids, AAV6 is identified to have the best transduction efficacy among the pooled AAV serotypes that were screened. We performed a single injection of 30 μL of AAV6-SpCas9 (Total 1.9 × 10^11^ vgs) suspension into the anterior chamber of two marmosets with a 30-gauge needle without touching the lens, iris, or other ocular tissue. The left eye is left untreated as control. The cornea tissues were harvested from the marmosets at 3 months post-treatment and the endothelial layer was removed via a Descemet peeling method. The remaining tissue bearing the epithelial and stroma layer were then assessed for AAV6 viral vector copies, SpCas9 mRNA and protein expression by qPCR and immunohistochemistry. Our results show that the AAV6 viral titer as well as the Cas9 mRNA expression level are significantly higher in the right cornea of both treated marmosets compared with the left control cornea, suggesting the successful transduction of the corneal epithelial cells by the AAV6-SpCas9 (Fig. 4A, B). In addition, we observed co-localization of the AAV capsid protein and SpCas9 protein with epithelial cell marker CK3 in the right treated cornea of the marmosets but not in the left control cornea by immunohistochemistry, which further confirms that the delivery of the SpCas9 gene cargo by AAV6 into the epithelial cells is highly efficient in the marmoset model (Fig. 4C, D). These results confirmed that the multiplex AAV single cell tropism assay identifies a suitable and efficient AAV serotype for use in gene delivery to the cell type of interest within a tissue or organ.

## Discussions

To date, most of the published AAV tropism assays utilize low-resolution methods to perform relative comparison between a few AAV serotypes, conducted either in vitro using homogenous cell lines or in vivo using bulk tissue organs. Advances in recent years have seen the development of single-cell assays or novel optical-image analysis tools to look at single AAV serotype tropism at higher resolution [38, 39]. In comparison, the assay that we have developed in this study can assess a larger panel of AAV serotypes simultaneously at single cells resolution within a single workflow. More recently, analytical pipelines involving separate library preparations and bioinformatics analysis tools were developed to enable the matching of the AAV barcodes to single cells identity to understand AAV tropism at single cell niche level [20, 21]. In this study, we demonstrated the use of a user-friendly platform that enables high-throughput multiplexing of AAV libraries for relative comparison of transduction efficiencies at single-cell resolution within a single library preparation and bioinformatics workflow. Our workflow required only the use of commercially available reagents to process both the cellular and viral barcodes simultaneously and is able to sequence both within a single library and run. For comparison, in Brown et al. and Ozturk et al., viral barcodes were amplified and prepared in a separate library preparation workflow, identified, and then matched to the cell barcodes with in-house bioinformatics. An overview of comparison between the pipelines can be found in Supplementary Table S6. Importantly, these prior studies have opened up opportunities for high-resolution AAV studies, but required sophisticated bioinformatics and separate library preparation processes that increase the barrier of entry. To enable technology uptake, our design and workflow integrated the transcriptomic data with the viral barcode data by using only the Cell Ranger software and the experiment requires only the simple use of a commercial kit.

With this simpler workflow, we evaluated the tropism of a library of AAV serotypes consisting of natural (AAV1, 2, 6, 7, 8, 9, and rh10) and engineered AAVs (DJ and Anc-80) for their transduction efficacy across different single-cell niches within the same organoid simultaneously. Engineered AAVs are particularly well-performing due to their prior engineering; AAV-DJ serotype was generated by DNA shuffling and stringently selected in both in vitro culture and mice [40]. Anc80 serotype was synthesized de novo via in silico reconstruction of evolutionary lineages of AAVs [41]. In this assay, we normalized the AAV barcodes via total expression, specifically via determining the relative distributions of AAV barcodes within each cell type. To identify the best AAV serotype that targets a given cell type, the transduction efficiency would directly correlate with the AAV barcode count. High-resolution quantification of every AAV serotype mRNA transcripts present in each cell reveals the AAV serotype(s) that has preferential tropism towards individual cell types. Although the current demonstration employs the use of 9 serotype variants, the assay supports substantially more variants as the barcoding strategy allows for exponential scaling up (i.e., the current 8-nt barcoding can support 65 K unique barcodes and serotypes, before implementation of error-tolerating or error-correcting encoding, while a 30-nt barcode would maximally support 10^18^). It is however important to note that the feasible library size would be lower than the theoretical limit, because sufficient representation of individual serotype variants would need to balance library size, tissue complexity, number of cells within the tissue, and transduction efficiency; Hence, depending on the tissue of interest, further studies might need to test the upper limit of feasible AAV library size and the viral titers needed to sufficiently transduce the cells.

As AAV tropism is largely driven by the binding between capsid and host surface receptor(s), differences in protein expression and orthologs among different species usually mean there will be tropism differences between the mouse model and human cell types. In this study, we selected human organoids as the in vitro model as they represent one of the closest models to human cells and tissues, although there are some limitations such as the presence of mainly precursor cells and absence of some mature cell types. For example, the AAV2 serotype is well established for retinal applications, but due to the absence of retinal cell types in the ocular organoids, we are unable to assess AAV tropism for retinal cells. Despite these biological limitations with organoids, the integrated bioinformatics pipeline for multiplex AAV single cells assay can also be applied to other tissues in culture or in vivo, since our technology counts AAV barcodes within single cell transcriptomes. Applying this technology on other complex tissue types in vivo might require titrating viral titers sufficient for transduction of cells, matching library size against the actual number of cells present in the tissue of interest for sufficient barcode representation, and complementing the sequencing readout with histological and in situ RNA assays.

To test the robustness of our assay, we further evaluated the efficacy of AAV serotype 6, which was identified to have the best transduction efficacy for ocular epithelial cell types, in the marmoset model. This result shares partial concordance with previous in vivo work that showed superior transduction performance of AAV6 for the mouse corneal endothelial layer and AAV8 transduction being excluded from the mouse endothelial and epithelial [42]. Our results confirmed the efficient transduction of the selected AAV6 and the successful expression of its cargo transgene in the epithelial layer of the marmoset corneas, validating the feasibility of using the assay for an initial high-throughput multiplex AAV screen in heterogenous cell types to identify the best viral vector for gene delivery into the cell-type(s)-of-interest. In treating corneal neovascularization, AAV-vectored anti-VEGF has been tested to be an efficacious, safe and longer-lasting therapy to replace current frequent doses of topical, intrastromal or subconjunctival administration [43]. The findings in this study that showed AAV6 has tropism for corneal epithelial cells may offer a more efficacious therapeutic delivery method via transduction of limbal epithelial cells in comparison to the intrastromal delivery by the AAV2 and AAV8-vectored anti-VEGF currently explored in another study [43–45]. This method can potentially be employed for clinical development by refining the selection of AAV serotypes for precise gene delivery to diseased tissues.

## Supplementary information

Supplementary Tables and Figures

Supplementary Data S1

Supplementary Data S2

Supplementary Data S3

## Data Availability

High-throughput sequencing data for both the bulk sequencing and single cell sequencing can be accessed via NCBI Sequence Read Archive database with SRA accession PRJNA742883 and BioProject accession code PRJNA742883. The in-house Python package that is used for bulk sequencing barcode counting can be found at the same accession ID.
