# Supplementary material for: Multiplex viral tropism assay in complex cell populations with single-cell resolution
Source: Gene Ther. 2022 Aug 23;29(9):555–65. doi: 10.1038/s41434-022-00360-3 (PMC9482877; doi:10.1038/s41434-022-00360-3)
Supplement: Supplementary file 1 — Supplementary Tables and Figures [file 41434_2022_360_MOESM1_ESM.docx]

**Tables**

**Table S1**

| Plasmids | Barcode | For barcoding |
| --- | --- | --- |
| pZac2.1-CMV-eGFP_A701 | ATCACGAC | AAV1 |
| pZac2.1-CMV-eGFP_A702 | ACAGTGGT | AAV2 |
| pZac2.1-CMV-eGFP_A706 | AACCCCTC | AAV6 |
| pZac2.1-CMV-eGFP_A707 | CCCAACCT | AAV7 |
| pZac2.1-CMV-eGFP_A708 | CACCACAC | AAV8 |
| pZac2.1-CMV-eGFP_A709 | GAAACCCA | AAV9 |
| pZac2.1-CMV-eGFP_A710 | TGTGACCA | AAV-rh10 |
| pZac2.1-CMV-eGFP_A711 | AGGGTCAA | AAV-DJ |
| pZac2.1-CMV-eGFP_A712 | AGGAGTGG | AAV-Anc80 |

**Table S2**

| Primers Name | Sequence |
| --- | --- |
| GFP_NGS_P7Amp | GTGACTGGAGTTCAGACGTGTGCTCTTCCGATCTGGGCATGGACGAGCTGTACAAG |
| GFP_NGS_P5Amp | ACACTCTTTCCCTACACGACGCTCTTCCGATCTGCAATGAAAATAAATTTCCTTTATTAGCCAACC |
| P5 Universal Primer | AATGATACGGCGACCACCGAGATCTACACTCTTTCCCTACACGACGCTCTTCCGATCT |
| P7 Barcode Adapter_UDI0001 | CAAGCAGAAGACGGCATACGAGATAGCGCTAGGTGACTGGAGTTCAGACGTGTGCTCTTCCGATCT |

**Table S3**

| AAV Barcodes | Bulk sequencing counts for ocular organoid (%) | Single cell sequencing counts for ocular organoid (%) |
| --- | --- | --- |
| A701 (AAV1) | 2.55 | 2.85 |
| A702 (AAV2) | 0.19 | 0.09 |
| A706 (AAV6) | 44.02 | 46.45 |
| A707 (AAV7) | 1.40 | 1.12 |
| A708 (AAV8) | 1.65 | 0.87 |
| A709 (AAV9) | 0.49 | 0.43 |
| A710 (AAVrh10) | 0.58 | 0.17 |
| A711 (AAVDJ) | 9.21 | 11.93 |
| A712 (AAVAnc80) | 39.88 | 36.07 |

**Table S4**

| AAV Barcodes | Bulk sequencing counts for cerebral organoid (%) | Single cell sequencing counts for cerebral organoid (%) |
| --- | --- | --- |
| A701 (AAV1) | 3.63 | 2.62 |
| A702 (AAV2) | 21.72 | 16.08 |
| A706 (AAV6) | 17.88 | 16.45 |
| A707 (AAV7) | 1.76 | 2.40 |
| A708 (AAV8) | 0.91 | 0.87 |
| A709 (AAV9) | 1.17 | 1.24 |
| A710 (AAVrh10) | 0.80 | 0.44 |
| A711 (AAVDJ) | 29.60 | 28.97 |
| A712 (AAVAnc80) | 22.33 | 30.93 |

**Table S5**

| Primers Name | Sequence |
| --- | --- |
| AAV_ITR_Fwd | GGAACCCCTAGTGATGGAGTT |
| AAV_ITR_Rev | CGGCCTCAGTGAGCGA |
| AAV_Probe | /56-FAM/CACTCCCTCTCTGCGCGCTCG/3HBQ_1/ |
| SpCas9N_Fwd | GGCGCCCTCCTGTTCGAC |
| SpCas9N_Rev | CCGTGCTGTTCTTTTGAGCCG |
| SpCas9N_Probe | /56-FAM/CGGGGAAACGGCCGAAGCCA/3BHQ_1/ |
| marGAPDH_Fwd | CGACAGACAGCCGCATC |
| marGAPDH_Rev | ACCTTCCCCATGGTGTCTC |
| marGAPDH_Probe | /56-FAM/TGTCTCCCATCGCCAGCCACATCC/3BHQ_1/ |

**Table S6**

| Single cell AAV tropism analysis pipelines | Keng et al. | Brown et al. | Ozturk et al. | Maturana et al. |
| --- | --- | --- | --- | --- |
| Single cell resolution for AAV tropism | Yes | Yes | Yes | Yes |
| Technical basis | Sequencing | Sequencing | Sequencing | Optical |
| Single step library preparation | Yes | Requires separate library preps | Requires separate library preps | Not applicable |
| Single step bioinformatics processing workflow | Yes | Requires separate bioinformatics processing | Requires separate bioinformatics processing | Not applicable |
| AAV library complexity demonstrated | ++ | +++ | +++ | + |
| Method complexity | + | ++ | ++ | +++ |

**Supplementary Figures**

**AAA**

GFP

NNNNNNNN

**A**

**Supplementary Figure S1. Design of AAV genomic cargo sequence for serotype barcoding and RNA transcripts capture for the modified 10X Cell Ranger pipeline for high-throughput single-cell analysis of AAV tropism.** (A) Schematic of design of AAV genomic cargo for capture and analysis of serotype barcodes. A mammalian promoter is selected for expression of a non-host protein in the human organoid cells. An eGFP transgene with barcode is expressed and can be distinguished from host gene transcripts. A unique 8 base-pair barcodes is included after the stop codon and before the polyadenylation tail, designed to be within the 98 bases from captured tail for Cell Ranger analysis. A polyadenylation tail sequence included for captured of RNA transcripts to the probes on 10X beads.


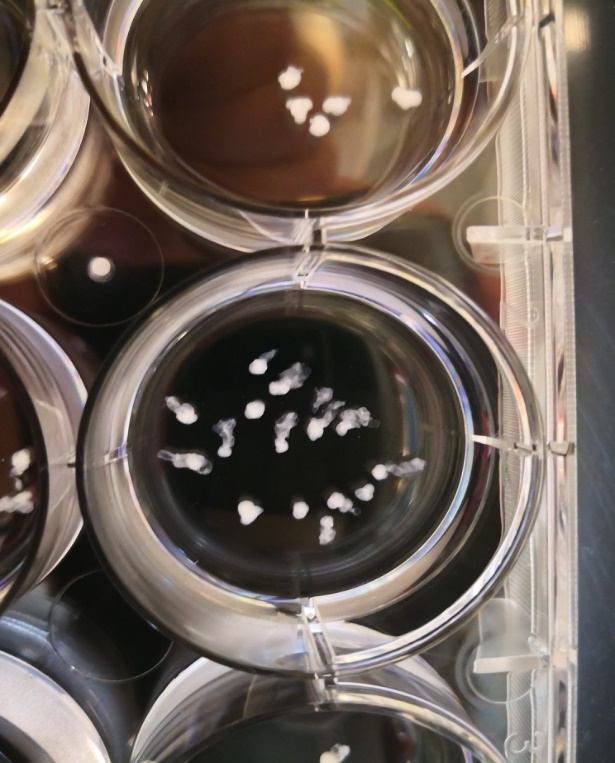

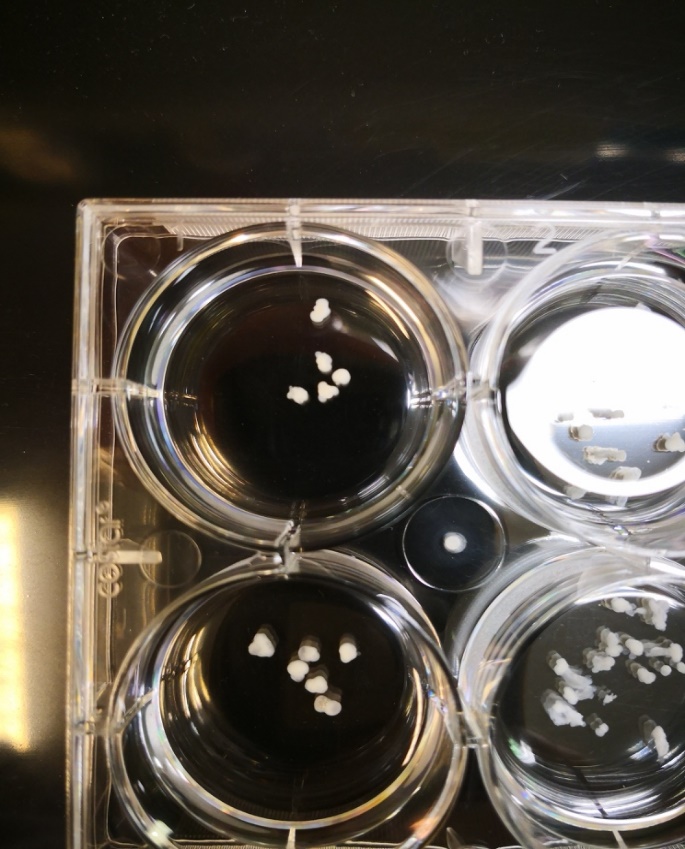

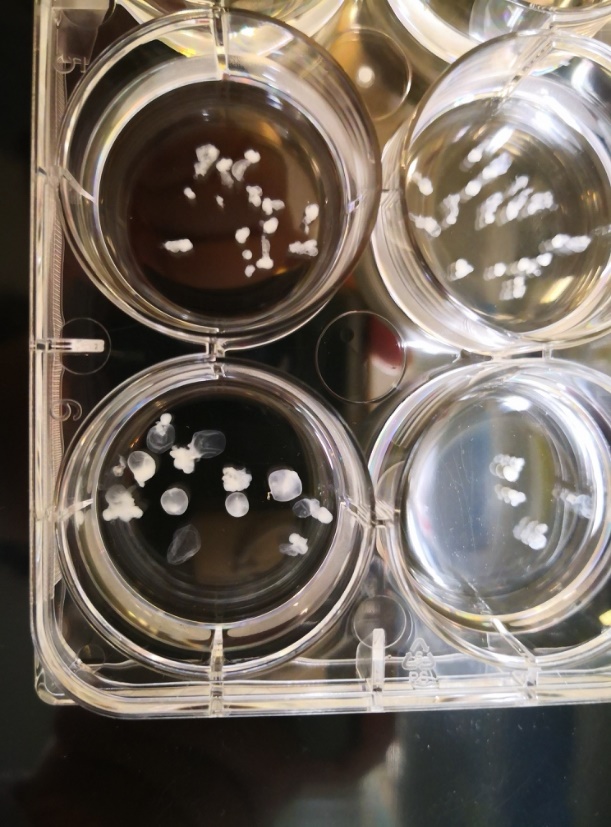

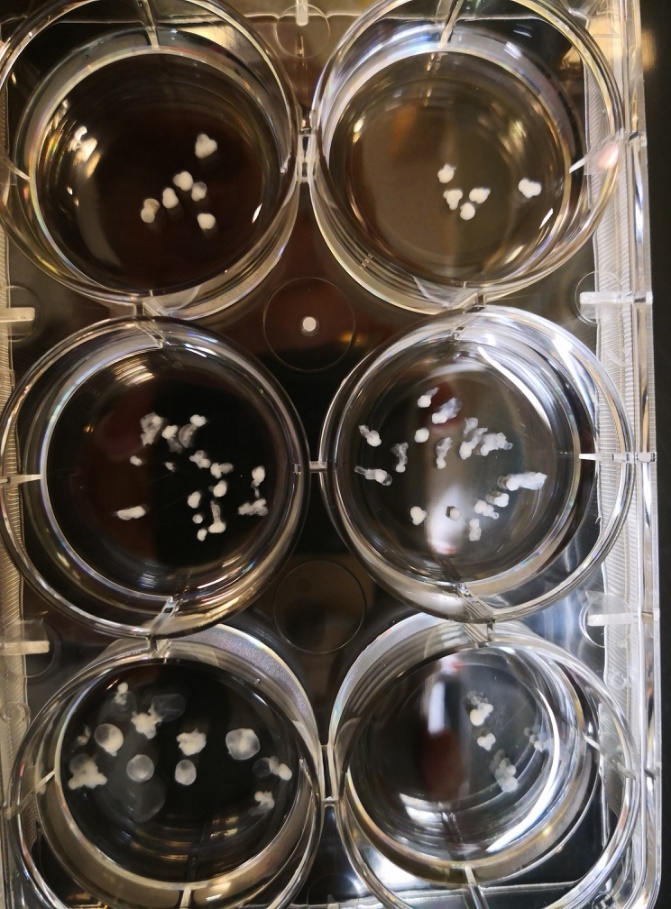


**Cerebral**

**Organoids**

**4 weeks**

**6 weeks**

**Ocular**

**Organoids**

**A**

**B**


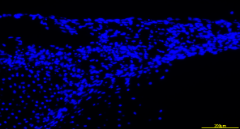

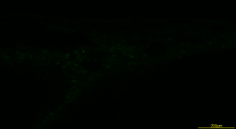

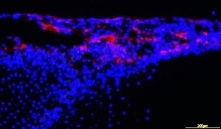

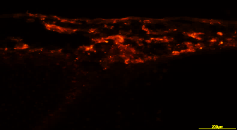


**DAPI**

**Merge**

**aSMA**

**CD31**


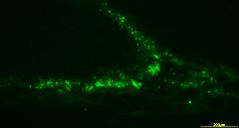

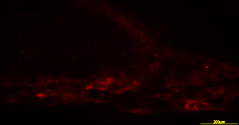

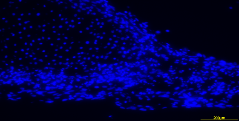


**DAPI**

**RAX**

**CHX10**


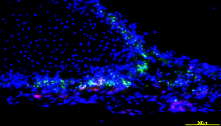


**Merge**


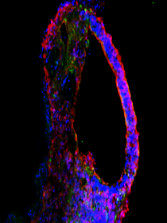

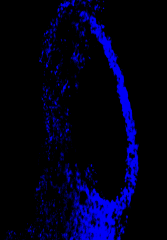

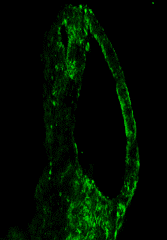

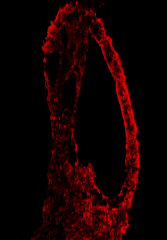

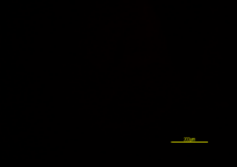

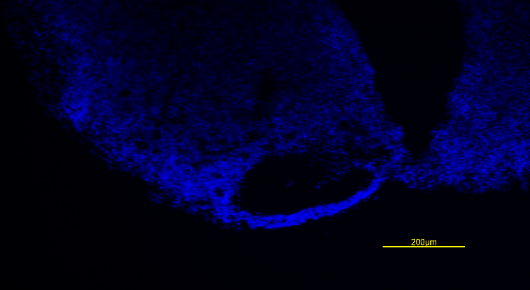

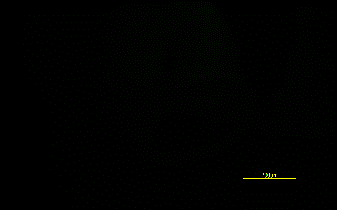

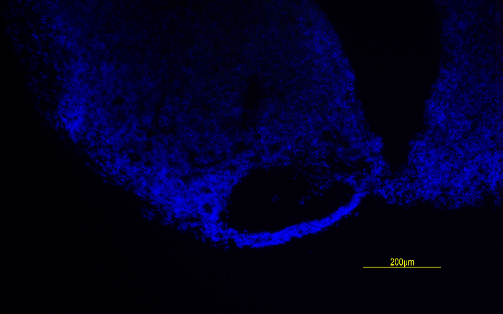


**Merge**

**Merge**

**DAPI**

**DAPI**

**PAX6**

**Neg**

**Neg**

**S100 β**

100um

100um

100um

100um

**C**


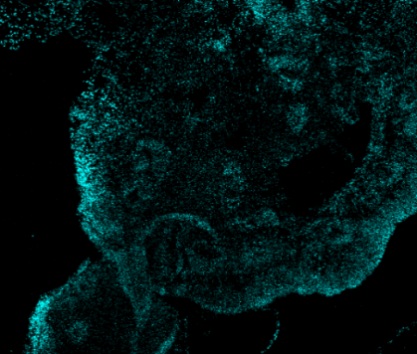

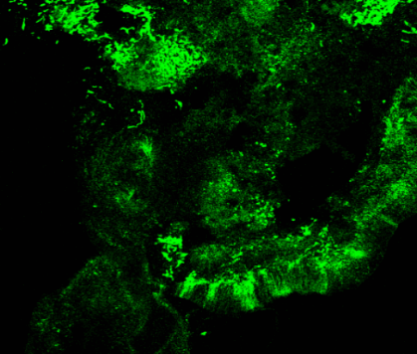

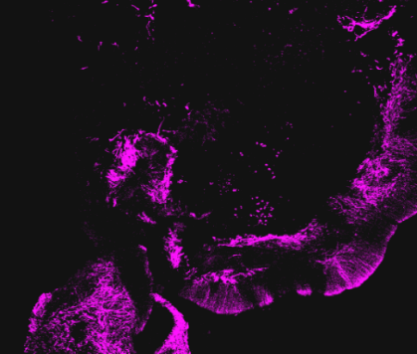

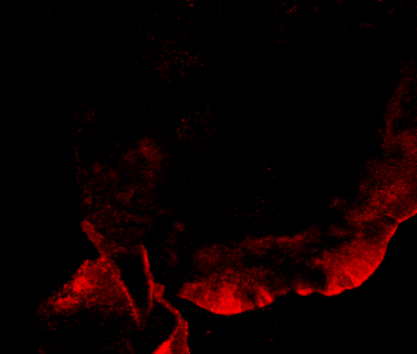

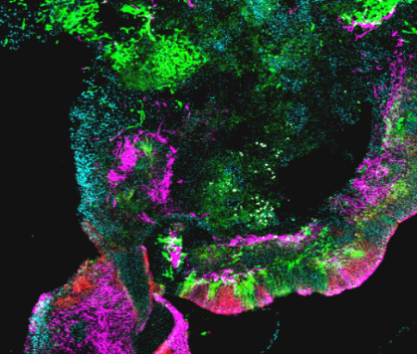


**DAPI**

**Merge**

**S100 β**

**NeuN**

**Map2**

200um


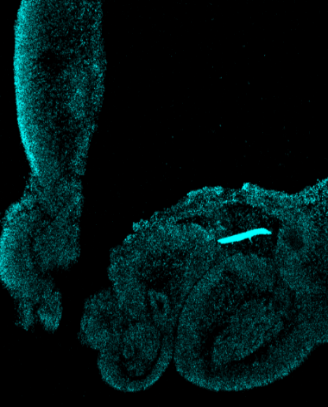

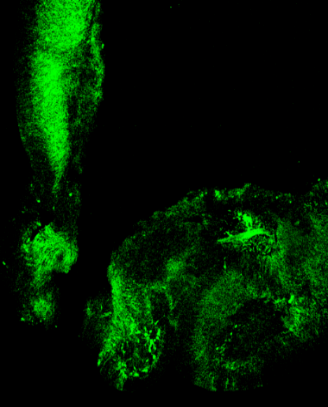

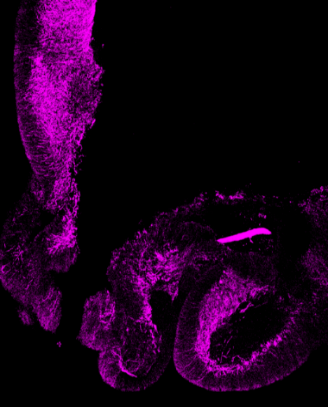

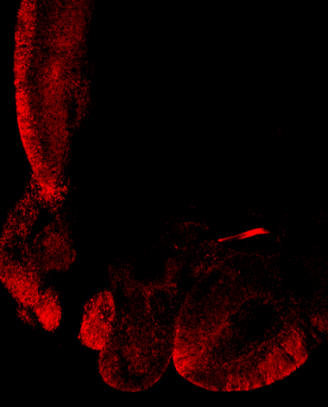

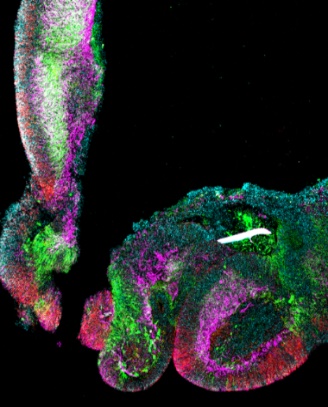


**DAPI**

**Merge**

**S100 β**

**NeuN**

**Map2**

200um

**Supplementary Figure S2. Ocular and cerebral organoids culture and characterization.** (A) Gross morphology of developing human cerebral and ocular organoids cultured for 6 weeks. Low magnification bright-field images revealed fluid-filled cavities of ocular (yellow arrow) and solid brain (blue arrow) organoids. (B) Histology sections of ocular organoids were stained for cellular markers for cell-type characterization. S100β – neuronal crest and developed ocular. PAX6 – ocular epithelial or endothelial cells. CHX10 – specification and morphogenesis of the sensory retina. RAX – developing eye and initial specification of retinal cells. CD31 - Schlemm’s canal endothelial. aSMA - trabecular meshwork and stroma. DAPI (49, 6-diamidino-2-phenylindole) stain for nuclei. Neg – negative control. (C) Histology sections of cerebral organoids sections were stained with for cellular markers for cell-type characterization. MAP2 – Positive in all neural cells. NeuN – Neuronal marker. S100 β – detect brain proteins and express in the neuronal cells. DAPI- stain for nuclei.

**A**

**
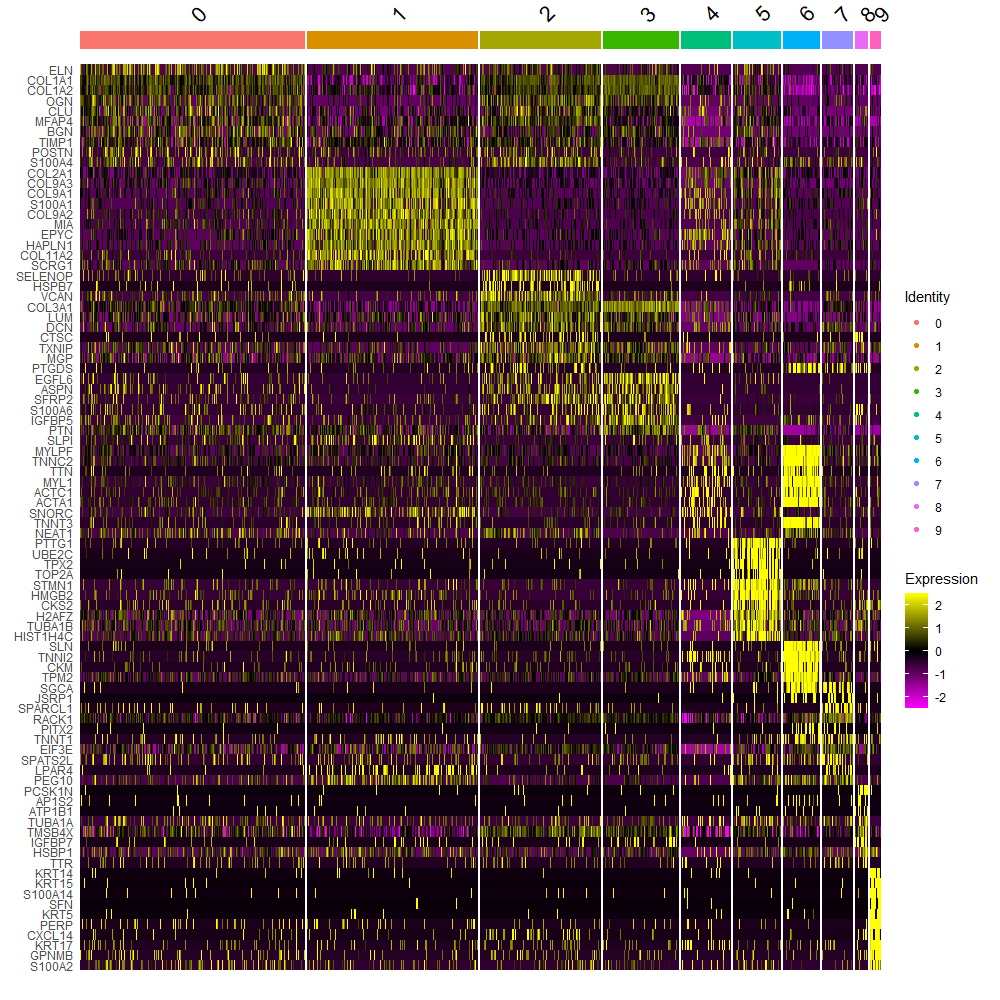
**

**B**

**
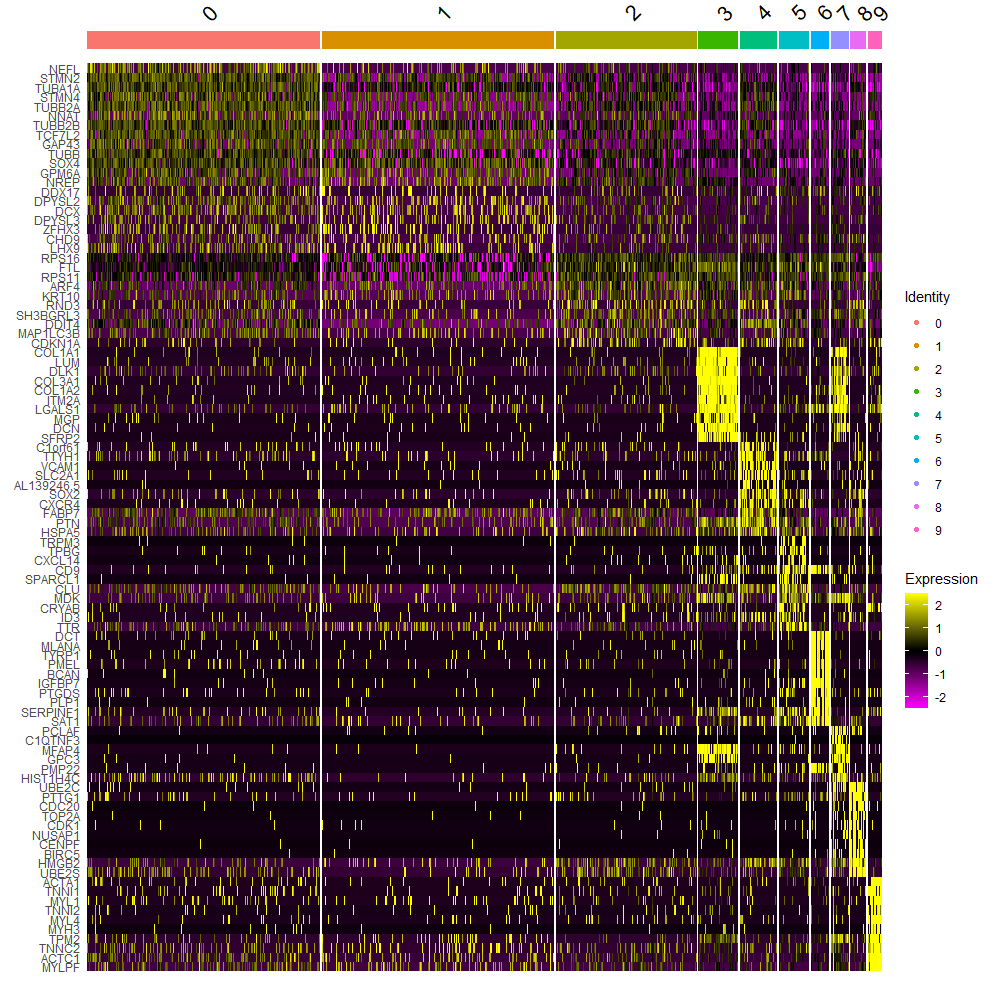
**

**C**

**
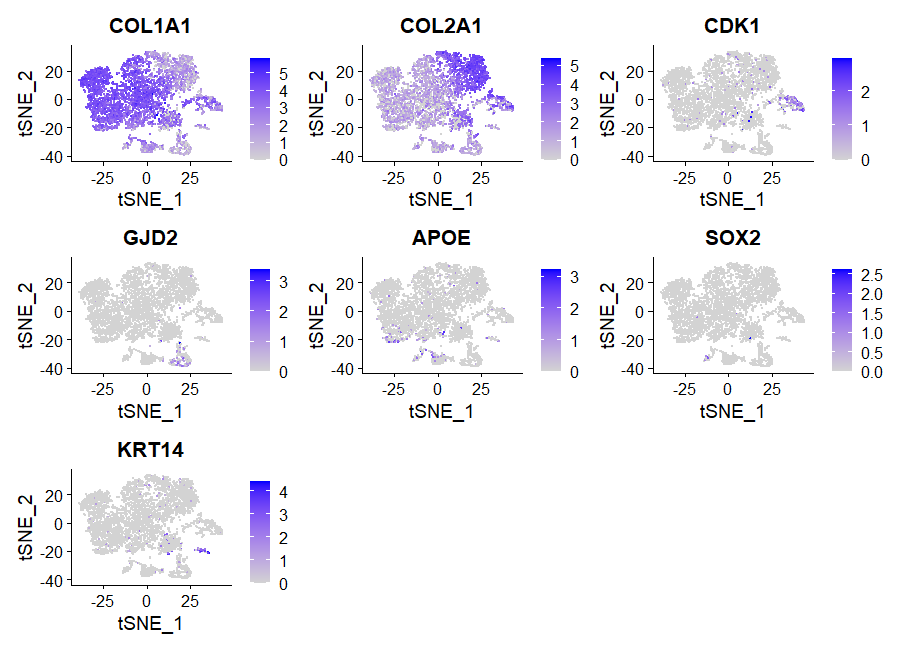
**

**D**

**
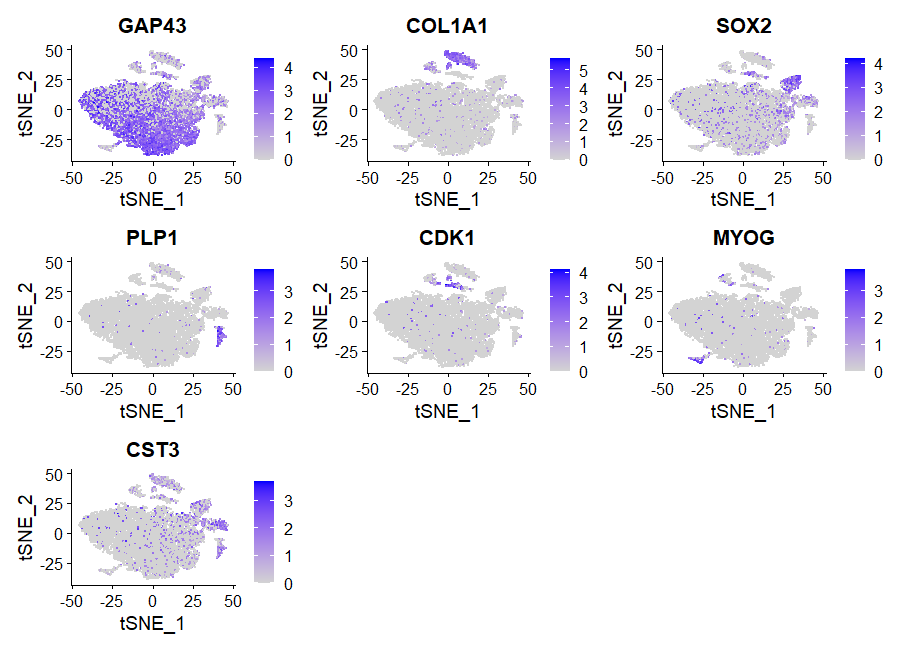
**

**Supplementary Figure S3. Single-cell RNA cluster annotations based on major known markers for different cell types in ocular and cerebral organoids.** (A) Heat map of expression profile for each cluster (0-9) in ocular organoid (B) Heat map of expression profile for each cluster (0-9) in cerebral organoid (C) Major gene markers for annotation of cell types in ocular organoid. Gene marker COL1A1 for fibroblast, COL1A2 for chondrocyte, CDK1 for dividing cells, GJD2 for amacrine, APOE for glia, SOX2 for neural stem cells (NSC) and KRT14 for cornea epithelial cells. (D) Major gene markers for annotation of cell types in cerebral organoid. Gene marker GAP43 for neuron, COL1A1 for fibroblast, SOX2 for neural stem cells (NSC), PLP1 for oligodendrocyte, CST3 for immature glial cells, CDK1 for dividing cells and MYOG for myocyte.


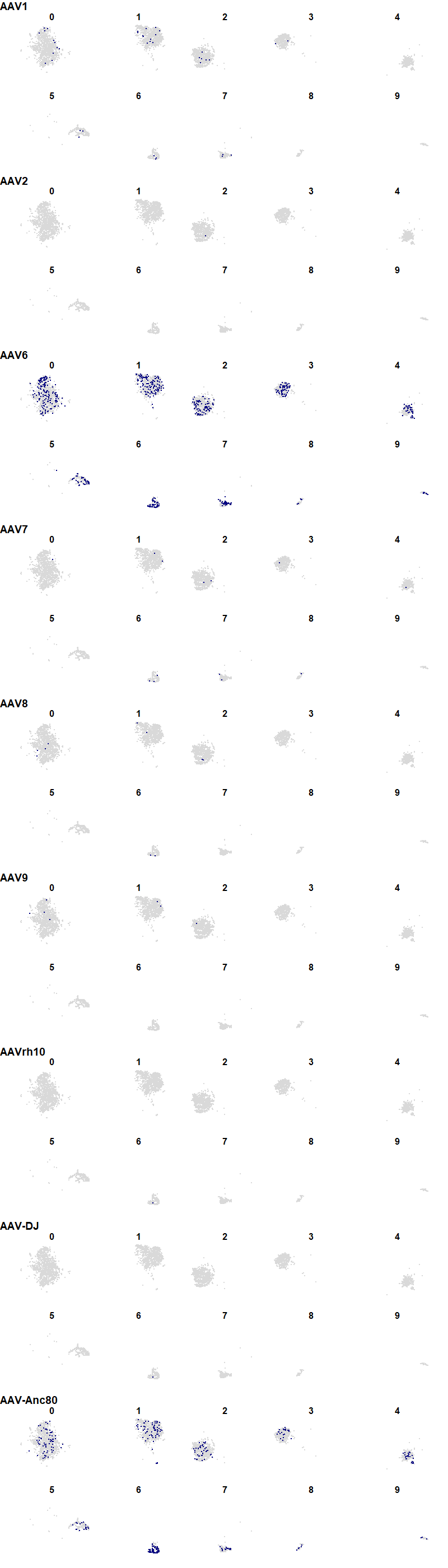


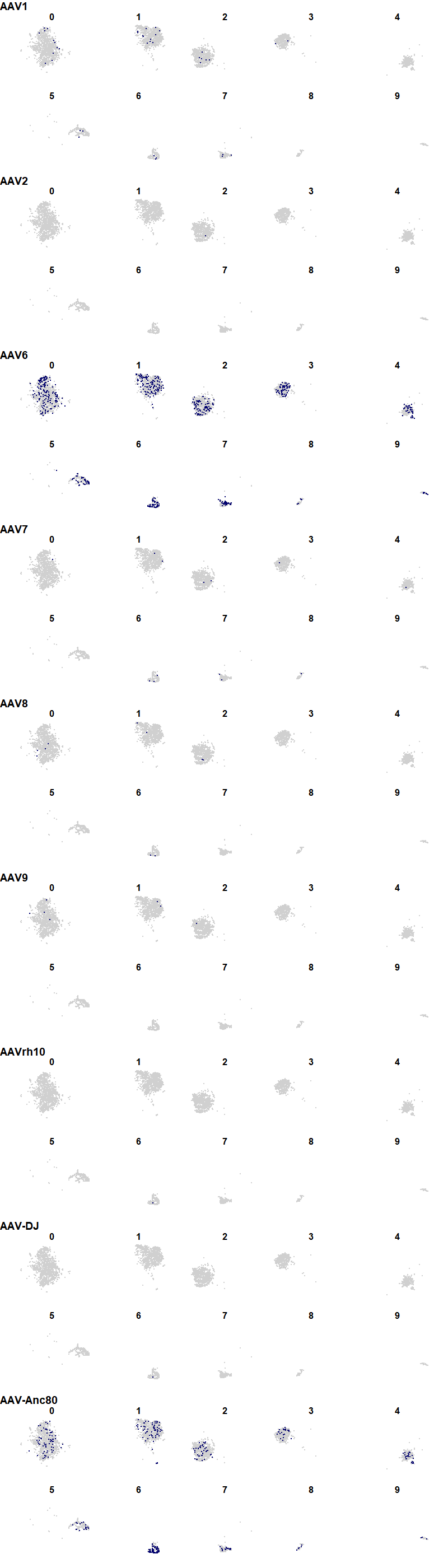


**Supplementary Figure S4.** t-SNE plots showing individual cells transduced with different AAV serotypes (each serotype represented by one plot), in each of the 10 clusters of the ocular organoid.


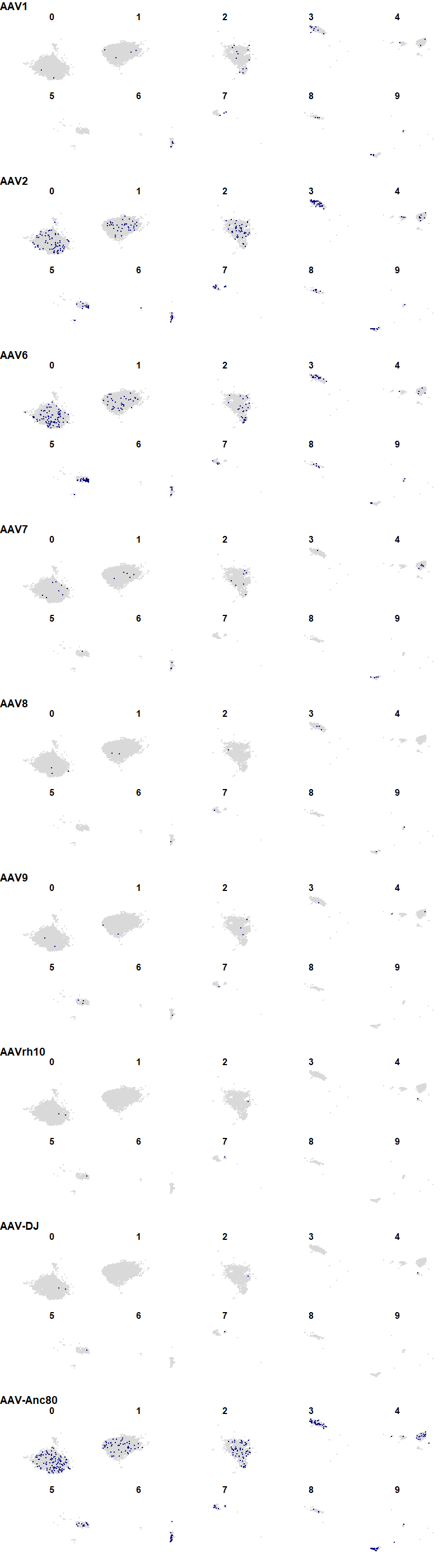


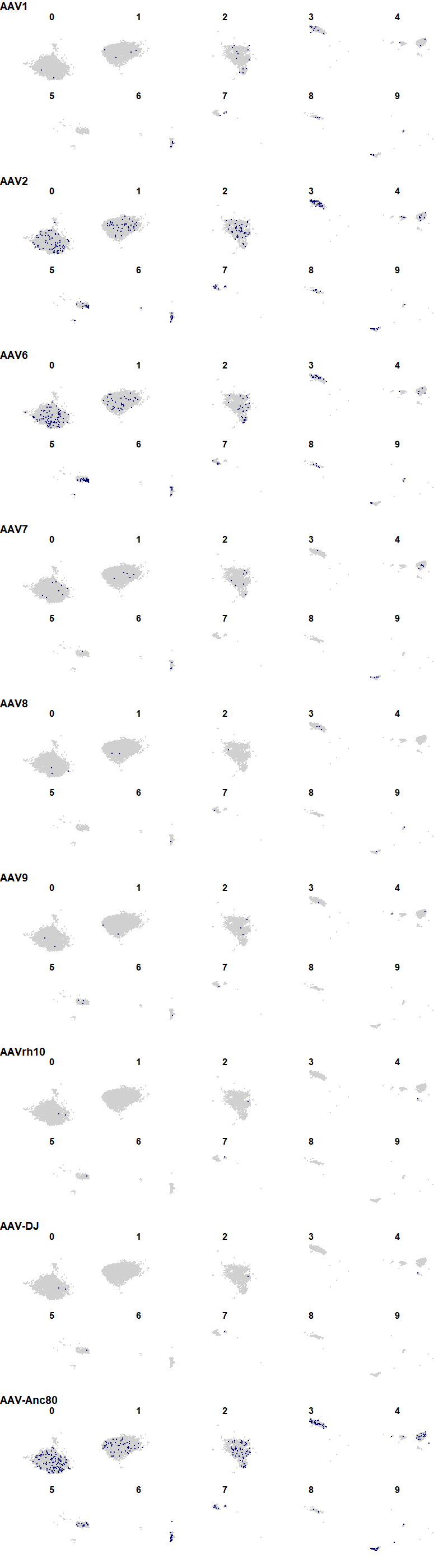


**Supplementary Figure S5.** t-SNE plots showing individual cells transduced with different AAV serotypes (each serotype represented by one plot), in each of the 10 clusters of the cerebral organoid.


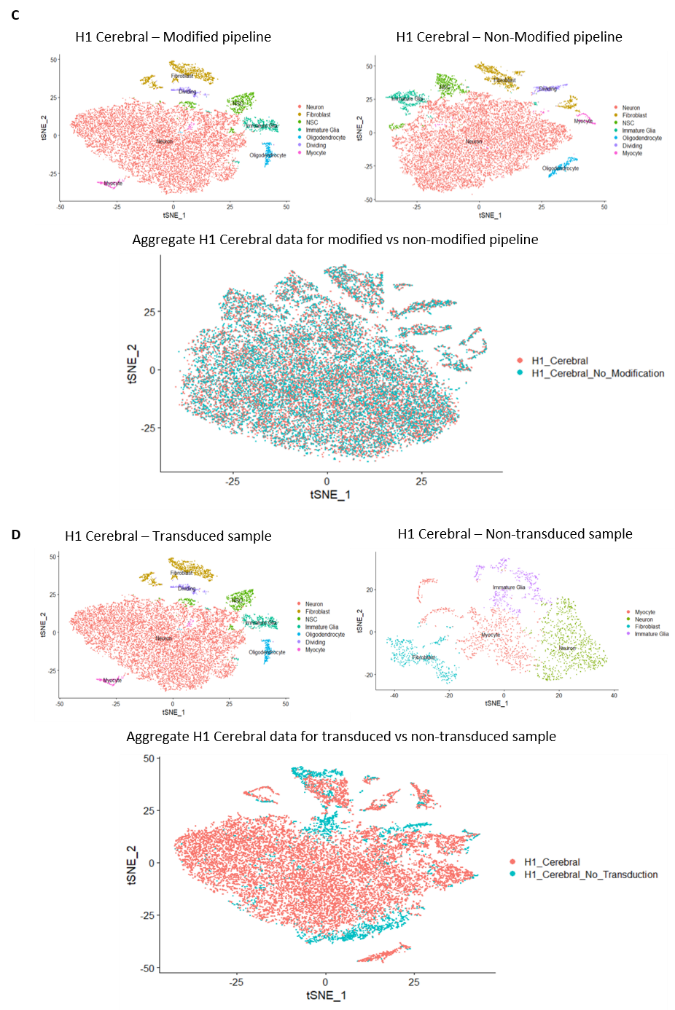

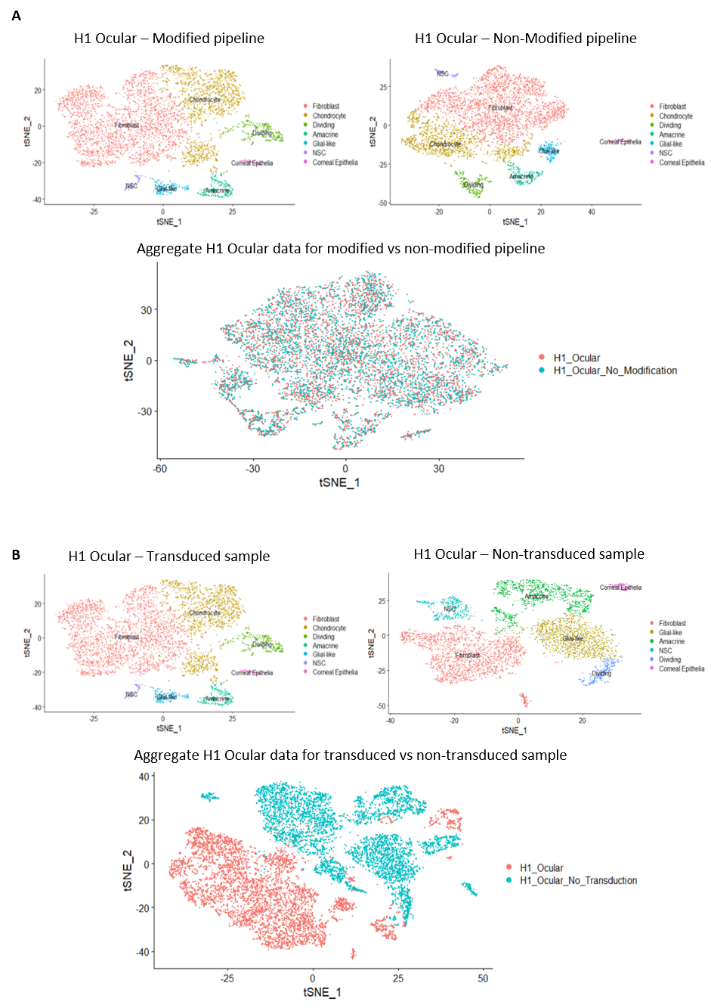


**Supplementary Figure S6.** (A) t-SNE plots of H1 ocular organoid data processed by modified pipeline including barcodes (top left) and non-modified pipeline excluding barcodes (top right) and aggregated together (bottom). (B) t-SNE plots of H1 ocular organoid transduced with AAV serotype panel (top left) and without transduction (top right) and aggregated together (bottom). (C) t-SNE plots of H1 cerebral organoid data processed by modified pipeline (top left) and non-modified pipeline excluding barcodes (top right) and aggregated together (bottom). (B) t-SNE plots of H1 cerebral organoid transduced with AAV serotype panel (top left) and without transduction (top right) and aggregated together (bottom).


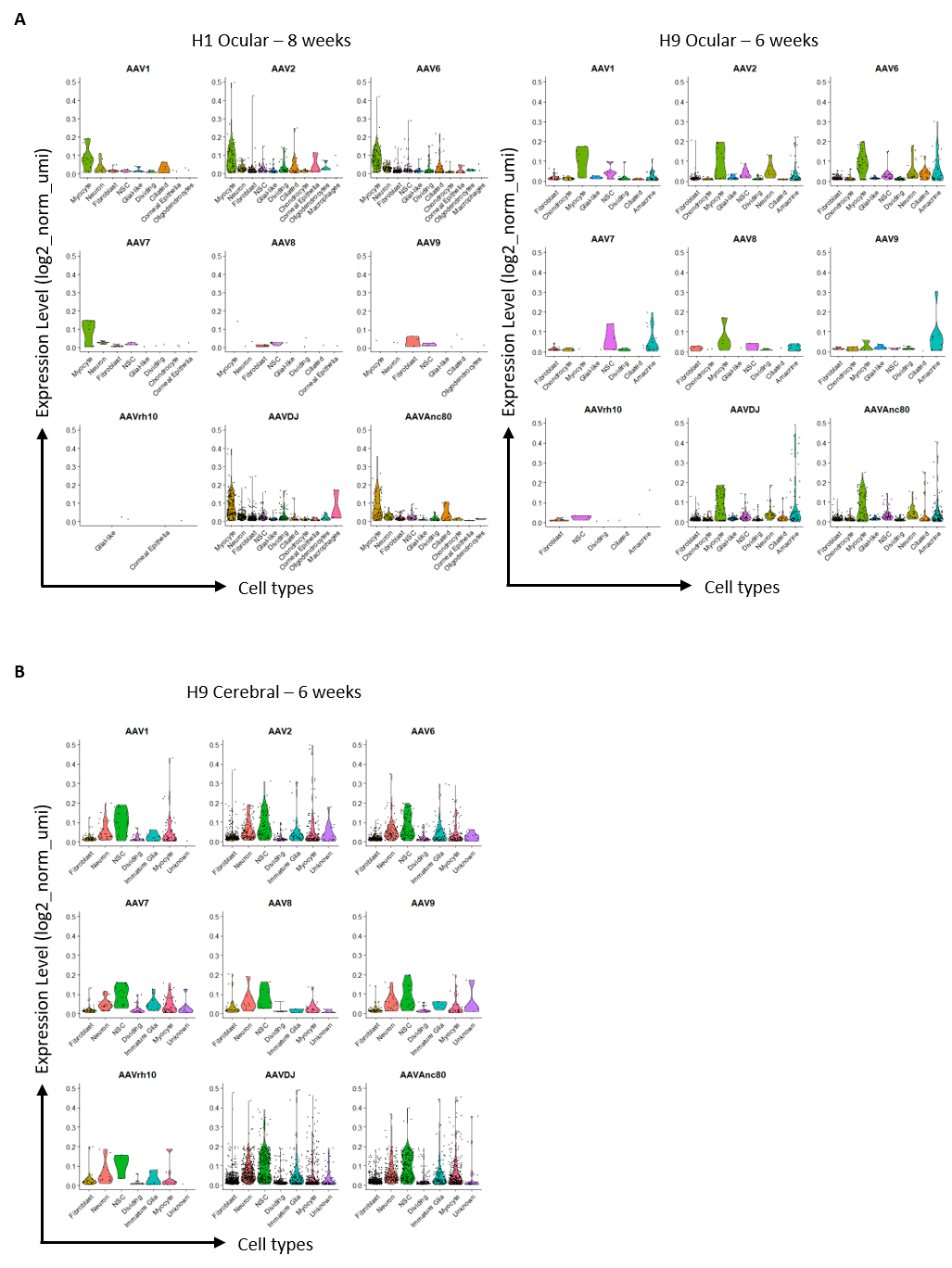


**Supplementary Fig. S7. AAV transduction of subpopulations in human organoids.** (A) AAV cell cluster tropism in transduced human ocular organoid cultured from H1 ES cells for 8 weeks. Data represent the cell counts that are transduced with each AAV serotype across the different cell types within the human ocular organoid. (B) AAV cell cluster tropism in transduced human ocular organoid cultured from H9 ES cells for 6 weeks. Data represent the cell counts that are transduced with each AAV serotype across the different cell types within the human ocular organoid. (C) AAV cell cluster tropism in transduced human cerebral organoid cultured from H9 ES cells cultured for 6 weeks. Data represent the cell counts that are transduced with each AAV serotype across the different cell types within the human cerebral organoid.
