# Supplementary material for: Multiplex viral tropism assay in complex cell populations with single-cell resolution
Source: Gene Ther. 2022 Aug 23;29(9):555–65. doi: 10.1038/s41434-022-00360-3 (PMC9482877; doi:10.1038/s41434-022-00360-3)
Supplement: Supplementary file 2 — Supplementary Data S1 [file 41434_2022_360_MOESM2_ESM.docx]

#move and extract tar files

for file in ./*.tar.gz; do tar -xvf $file -C ./XMP; done

#have to rename all fastq files to the convention that 10x cellranger recognises

cd/mnt/volume1/fastq

rename 's/HS007-PE-R00353/S1/' *.gz

rename 's/_unaligned//' *.gz

#To export path for Cellranger program

export PATH=/mnt/volume1/Cellranger/cellranger-3.0.1:$PATH

#Ensure fasta file is in fa format and in the same folder as genome.fa file.

$ cat mygene.fa >> genome.fa

**#To modify GTF file**, go to the gtf file folder.

$ nano genes.gtf

#Alt / to go to the end of the page

#Add in the information (see below).

#Ctlr X to exit and save file.

GFP1 me exon 1 19 . + . gene_id "GFP1"; transcript_id "GFP1";

GFP2 me exon 1 19 . + . gene_id "GFP2"; transcript_id "GFP2";

GFP3 me exon 1 19 . + . gene_id "GFP3"; transcript_id "GFP3";

GFP4 me exon 1 19 . + . gene_id "GFP4"; transcript_id "GFP4";

GFP5 me exon 1 19 . + . gene_id "GFP5"; transcript_id "GFP5";

GFP6 me exon 1 19 . + . gene_id "GFP6"; transcript_id "GFP6";

GFP7 me exon 1 19 . + . gene_id "GFP7"; transcript_id "GFP7";

GFP8 me exon 1 19 . + . gene_id "GFP8"; transcript_id "GFP8";

GFP9 me exon 1 19 . + . gene_id "GFP9"; transcript_id "GFP9";

GFP10 me exon 1 19 . + . gene_id "GFP10"; transcript_id "GFP10";

GFP11 me exon 1 19 . + . gene_id "GFP11"; transcript_id "GFP11";

GFP12 me exon 1 19 . + . gene_id "GFP12"; transcript_id "GFP12";

GFP me exon 1 60 . + . gene_id "GFP"; transcript_id "GFP";"eGFP1c";

**#To modify the genome file**, go into the reference folder that contains the genome file and key in the command lines below:

$ nano genome.fa

#Alt/ to go to end of page

#Add in the information (see below).

#Ctlr X to exit and save file

#Insert the lines below:

>GFP1

TAAATCGATCGATCACGAC

>GFP2

TAAATCGATCGACAGTGGT

>GFP3

TAAATCGATCGCAGATCCA

>GFP4

TAAATCGATCGACAAACGG

>GFP5

TAAATCGATCGACCCAGCA

>GFP6

TAAATCGATCGAACCCCTC

>GFP7

TAAATCGATCGCCCAACCT

>GFP8

TAAATCGATCGCACCACAC

>GFP9

TAAATCGATCGGAAACCCA

>GFP10

TAAATCGATCGTGTGACCA

>GFP11

TAAATCGATCGAGGGTCAA

>GFP12

TAAATCGATCGAGGAGTGG

>GFP

gacgagctgtacaagtaaTAATAAATCGATCGNNNNNNNNaccggttggctaataaagga

**#After editing genes.gtf and genome.fa files, go into the genome folder with the edited genome.fa file and proceed to make new reference folder by typing in the command below:**

cellranger mkref --genome=EditedReference --fasta=genome.fa --genes=path to edited genes.gtf

#Note --genome= is not a path, state a new folder for the new reference file.

#Note –fasta= state the new edited genome.fa name

**#To perform the cell count using modified genome.fa and modified gtf files:**

Usage:

count

--id=ID

[--fastqs=PATH]

[--sample=PREFIX]

--transcriptome=DIR

[options]

count <run_id> <mro> [options]

count -h | --help | --version

#Define path. Example

export PATH=/mnt/volume1/Cellranger/cellranger-3.0.1:$PATH

#Perform count. Examples.

nohup cellranger count \

--id=OcularPoolAAVcount1 \

--description=OcularAAVcount1 \

--transcriptome=/mnt/volume1/Cellranger/refGFP-cellranger-GRCh38-3.0.0 \

--fastqs=/mnt/volume1/scOcularOrganoid \

--sample=XHE002-ACAGAGGT,XHE003-TATAGTTG,XHE004-CGGTCCCA,XHE005-GTCCTAAC \

--expect-cells=10000 &

nohup cellranger count \

--id=CerebralAAVcount1 \

--description=CerebralAAVcount1 \

--transcriptome=/mnt/volume1/Cellranger/refGFP-cellranger-GRCh38-3.0.0 \

--fastqs=/mnt/volume1/scCerebralOrganoid \

--sample=XHE024 \

--expect-cells=10000 &
